# Genome‐wide analysis of the callose enzyme families of fertile and sterile flower buds of the Chinese cabbage (*Brassica rapa* L. ssp. *pekinensis*)

**DOI:** 10.1002/2211-5463.12685

**Published:** 2019-07-12

**Authors:** Yanan Pu, Lingyun Hou, Yingqi Guo, Ikram Ullah, Yongping Yang, Yanling Yue

**Affiliations:** ^1^ College of Landscape and Horticulture Yunnan Agricultural University Kunming China; ^2^ Kunming Institute of Botany Chinese Academy of Sciences Kunming China; ^3^ University of Chinese Academy of Sciences Beijing China; ^4^ Kunming Institute of Zoology Chinese Academy of Sciences Kunming China

**Keywords:** callose, Chinese cabbage, enzyme family, gene expression, nuclear sterility

## Abstract

Callose is a β‐1,3‐glucan commonly found in higher plants that plays an important role in regulating plant pollen development. It is synthesized by glucan synthase‐like (GSL) and is degraded by the enzyme endo‐1,3‐β‐glucosidase. However, genome‐wide analyses of callose GSL and endo‐1,3‐β‐glucosidase enzymes in fertile and sterile flower buds of Chinese cabbage have not yet been reported. Here, we show that delayed callose degradation at the tetrad stage may be the main cause of microspore abortion in Chinese cabbage with nuclear sterility near‐isogenic line ‘10L03’. Fifteen callose GSLs and 77 endo‐1,3‐β‐glucosidase enzymes were identified in Chinese cabbage. Phylogenetic, gene structural and chromosomal analyses revealed that the expansion occurred due to three polyploidization events of these two gene families. Expression pattern analysis showed that the GSL and endo‐1,3‐β‐glucosidase enzymes are involved in the development of various tissues and that the genes functionally diverged during long‐term evolution. Relative gene expression analysis of Chinese cabbage flowers at different developmental stages showed that high expression of the synthetic enzyme *BraA01g041620* and low expression of *AtA6*‐homologous genes (*BraA04g008040*,* BraA07g009320*,* BraA01g030220* and *BraA03g040850*) and two other genes (*BraA10g020080* and *BraA05g038340*) for degrading enzymes in the meiosis and tetrad stages may cause nuclear sterility in the near‐isogenic line ‘10L03’. Overall, our data provide an important foundation for comprehending the potential roles of the callose GSL and endo‐1,3‐β‐glucosidase enzymes in regulating pollen development in Chinese cabbage.

**Abbreviations**
CMS: cytoplasmic male sterility
GMS: genetic male sterility
GRAVY: grand average of hydrophobicity
GSL: glucan synthase‐like
NIL: near‐isogenic line
pI: isoelectric point
TMH: transmembrane helix

Pollen development is an extremely important biological process in flowering plants and an indispensable life process for plant genetic breeding 1. Pollen development abnormalities make the formation of functional pollen difficult, leading to male sterility, which seriously affects the reproduction and evolution of plants 2. As the utilization of crop heterosis continues to increase, male sterility has become more widely used in crop breeding practices to make it easier to achieve heterosis in crops that use hybrid pollination, which can greatly increase crop yields and resistance 3. Chinese cabbage has two types of male sterility: cytoplasmic male sterility (CMS) and genetic male sterility (GMS) 4. Previous studies 5 have shown that CMS is the result of gene interaction between the nuclear and cytoplasmic genomes, and the interaction between the nuclear genome and mitochondrial genome leads to nuclear–cytoplasmic inconsistency, thus disturbing the normal metabolic process 6. The main cytological characteristic is tapetum cell death, and the physiological response is the disorder of mitochondrial energy metabolism. GMS inheritance is relatively simple, generally controlled by one or several pairs of nuclear genes, and has nothing to do with cytoplasmic genes. But a population of 100% male sterile plants could not be found when a test cross was employed in selecting its maintainer line, whether GMS is controlled by dominant or recessive genes 7. The male sterility could be maintained at only 50% by the sibling in an AB line in which the separation ratio of sterile and fertile plants is 1 : 1. There are two kinds of AB lines, AB line 1 and AB line 2. Twenty‐five percent of the progeny from selfing of male fertile plants in an AB line 1 are male sterile, and all progeny from selfing of male fertile plants in an AB line 2 are male fertile 8. Fertile plants must be identified and rogued before flowering when AB lines are utilized to produce hybrid seed, which is more costly and labor intensive. GMS lines with a 100% sterility rate were obtained for the first time by crossing between male sterile plants in AB line 1 and male fertile plants in AB line 2 by Zhang *et al*. in 1990 9. In 1996, the multiple allele hypothesis was raised to explain the inheritance of this male sterility, which was controlled by three alleles at a single locus including male sterile allele ‘Ms’, male fertile allele ‘ms’ and a restorer allele ‘Ms^f^’. The dominant–recessive relationship of these three alleles was Ms^f^>Ms>ms^10^. Recessive GMS is sensitive to environment, while dominant GMS is insensitive to environment 11. Compared with CMS, GMS has the advantages of good stability, incompleteness, no negative impact on cytoplasm, and wide distribution of fertility restoration genes. Studies of the mechanism of nuclear sterility in *Brassica napus* 12, tobacco (*Nicotiana tabacum* L.) 13, 14 and wheat (*Triticum aestivum* L.) 15 have shown that most plant sterility originates from the abnormal development of pollen at different stages. Because of the significant production and theoretical research value of male sterility, it has long been a subject of interest. However, pollen development is a very complex process involving the regulation and expression of many genes. Mutation or abnormal expression of any of the key genes may cause abnormal pollen development and lead to male sterility. In‐depth studies of pollen development‐related genes and their molecular regulatory mechanisms will provide an important theoretical foundation for understanding plant male sterility and creating superior male sterile lines.

Callose is an important feature of sexual reproduction in plants, especially in sexual cells that are about to divide, as it forms the mother cell wall and the zygote wall of large spores 16, and plays a protective role by providing mechanical isolation, a chemical barrier, or a molecular sieve, and prevents cell aggregation 17. Callose prevents agglomeration and fusion between cells when microspores are released, protects developing microspores from premature expansion and rupture, and acts as a ‘mold’ for the development and shaping of pollen outer wall patterns 16, 18. The callose of the external body wall of the plant anther tetrad plays an important role in the development and maturation of the microspores 13. Decomposition at the proper time is critical; premature callose decomposition leads to incomplete growth of the microspores, and a lack of decomposition or delayed decomposition results in problems with the normal release of the microspores 14, 19, which can lead to pollen sterility.

Studies on callose have shown that the main component is β‐1,3‐glucan, which is synthesized by the enzyme glucan synthase‐like (GSL) and degraded by endo‐1,3‐β‐glucosidase enzymes 20, 21. Twelve GSL genes (*AtGSL1–AtGSL12*) have been found in *Arabidopsis* that are involved in the synthesis of callose 22. *AtGSL2* is a major callose synthesis gene in pollen development 23. Knock‐out mutation of *AtGSL2* has little effect on vegetative growth, but severely affects pollen development and callose deposition in pollen tube germination, resulting in the callose wall being completely absent and collapse of the pollen wall 24. Overexpression of *AtGSL2* in *Arabidopsis* resulted in abnormal deposition of callose during microsporogenesis and promoted early release of pollen before flowering 25. *AtGSL1* and *AtGSL5* are not required for callose synthesis in pollen mother cells, but are essential for the synthesis of tetrad callose. The mutant *gsl1‐1*/+*gsl5‐2*/*gsl5‐3* exhibited tetrad callose depletion, tetrad malformation, and incomplete isolation of microspores 26. *AtGSL10* and *AtGSL8* cause callose deposition on cell plates, cell walls and intercellular filaments. The *AtGSL8* mutant shows delayed callose deposition at the cell plate and suffers from cytokinesis defects that cause seedling death 27. The microspores of *AtGSL10* and *AtGSL8* mutants are unable to enter mitosis, which eventually leads to male gametophyte death 28. In rice, the *OsGSL5* gene plays a regulatory role in the late meiotic stage. In *OsGSL5* mutants, the callose synthesis catalyzed by GSL5 is greatly reduced, and the development of mitotic and tetrad abnormalities leads to a decrease in the fertility of the mutant plants 29. Plant anther endo‐1,3‐β‐glucosidase genes have currently only been cloned in *Arabidopsis* (*AtA6*) and *B. napus* (*BnA6*) and are localized in tapetum cells 21. Endo‐1,3‐β‐glucosidase enzymes are highly expressed just before the disintegration of the tetrads and then disappear rapidly 18. This shows that endo‐1,3‐β‐glucosidase enzymes play an important role in the development of anthers in plants and are closely related to the development and maturation of male gametes. However, to our knowledge, a genome‐wide analysis of callose GSL and endo‐1,3‐β‐glucosidase enzymes in fertile and sterile flower buds of Chinese cabbage has not yet been reported.

In this study, we observed callose degradation by paraffin sections and light microscopy, then we identified 15 callose GSL and 77 endo‐1,3‐β‐glucosidase enzymes in Chinese cabbage by genome‐wide analysis of the callose enzyme families. Furthermore, we performed phylogenetic, gene structure, chromosomal location, expression profiling, and relative expression analyses. Our results provide an important foundation for understanding the potential roles of callose GSL and endo‐1,3‐β‐glucosidase enzymes in regulating pollen development in Chinese cabbage.

## Materials and methods

### Plant materials

Chinese cabbage nuclear sterility near‐isogenic line (NIL) ‘10L03’ was used in this study. It was bred from saturated backcross progenies of the inbred ‘02s005’ (backcross parent, Ms^f^Ms^f^ genotype) and the male sterile line (Msms genotype). Genotypes of fertile and sterile plants are Ms^f^Ms and MsMs respectively in ‘10L03’. Siblings between sterile and fertile plants of ‘10L03’ could reproduce, and sterile and fertile plants in their progeny maintain 1 : 1 separation. ‘10L03’ was cultivated in the experimental plot in Yunnan Agriculture University (Kunming, Yunnan Province, China). Fertility of plants was identified when flowering. Buds from fertile and sterile plants were collected for paraffin embedding. With regard to *Arabidopsis* pollen development 30, stages were identified and microspore developmental stages of different bud sizes were investigated by microscopy. Fertile and sterile buds at meiosis (stage 6, fertile: 1.40 × 1.10 mm, sterile: 1.30 × 1.00 mm), tetrad (stage 7, fertile: 2.10 × 1.30 mm, sterile: 2.00 × 1.20 mm), mononuclear (stage 8, fertile: 2.80 × 1.60 mm, sterile: 2.60 × 1.40 mm), and mature (stage 13, fertile: 6.00 × 2.70 mm, sterile: 5.30 × 2.20 mm) were chosen and stored at −80 °C for RNA extraction.

### Paraffin sectioning

Fertile and sterile buds were fixed in FAA fixative solution (every 100 mL of which contains 90 mL of 50% ethanol, 5 mL of acetic acid, and of 5 mL formalin) for 24 h at room temperature, which was followed by dehydration in increasing grades of ethanol. Ethanol and xylene in a volume ratio of 2 : 1, 1 : 1 and 1 : 2 were used for clearing, and the samples were embedded in paraffin. Paraffin sections were cut to a thickness of 8 μm on an semi‐automatic microtome (YD‐335, Jinhua Yidi Medical Equipment Co., Ltd., Jinhua, China) followed by dewaxing with xylene. Sections were stained with haematoxylin and eosin, visualized with a Leica DM2000 (Beijing, China) optical microscopy and photographed.

### Aniline blue staining for callose

For callose staining, transverse anther sections and microspores released from the anthers were stained with 0.1% aniline blue solution in 0.077 m phosphate buffer (pH 8.5) 31 for about 2 h at room temperature and visualized using a Leica fluorescence microscope.

### Identification of the callose GSL and endo‐1,3‐β‐glucosidase enzyme families in Chinese cabbage

The coding DNA sequences of 12 *Arabidopsis* callose GSL enzymes and 25 endo‐1,3‐β‐glucosidase enzymes were downloaded from The Arabidopsis Information Resource (www.arabidopsis.org) and used as queries in blast searches against the *Brassica rapa* genomes (http://brassicadb.org/brad/datasets/pub/BrassicaceaeGenome/Brassica_rapa/V3.0/). Each protein with its domains and functional sites was examined with smart (http://smart.embl-heidelberg.de/). All callose GSL protein sequences containing the Glucan_GSL domains (PF02364) and all callose endo‐1,3‐β‐glucosidase protein sequences with the Glyco_hydro_17 motif (PF00332) were extracted as candidates.

The GenBank non‐redundant protein database was used to search against the candidates. dnaman software (LynnonBiosoft, San Ramon, CA, USA) was used for the homology analysis between *B. rapa* and *Arabidopsis*. wolf psort (http://wolfpsort.org) 32 was used to predict protein subcellular localization. The tmhmm server (http://www.cbs.dtu.dk/services/TMHMM/) was used to estimate the number of transmembrane helical (TMHs) domains. The molecular mass, theoretical pI and grand average of hydropathicity (GRAVY) were calculated using the tool of expasy 33 (http://web.expasy.org/protparam/).

### Phylogenetic analyses of the callose GSL and endo‐1,3‐β‐glucosidase enzymes

The full‐length Chinese cabbage callose GSL and endo‐1,3‐β‐glucosidase protein sequences were aligned using the program mafft 7.0, and phylogenetic reconstruction used the neighbor‐joining method with mega7 software 34. Bootstrap values for each branch were estimated (with 1000 replicates) to assess the relative support. A diagram of the intron/exon structures of the callose GSL and endo‐1,3‐β‐glucosidase genes was generated using the online Gene Structure Display Server (http://gsds.cbi.pku.edu.cn/) 35. Subsequently, the meme program was used to search for conserved motifs in the *B. rapa* callose GSL and endo‐1,3‐β‐glucosidase protein sequences 36.

### Chromosomal locations and gene structures of the callose GSL and endo‐1,3‐β‐glucosidase enzymes

To investigate the callose GSL and endo‐1,3‐β‐glucosidase gene locations, a map of their distribution throughout the *B. rapa* genome (version 3.0) was drawn with the mapinspect tool (http://www.plantbreeding.wur.nl/UK/software_mapinspect.html).

### Expression profiles of callose GSL and endo‐1,3‐β‐glucosidase genes in *B. rapa*

Transcriptome data for four tissues in *B. rapa* were obtained from previous research 37 and reanalyzed. From the samples were obtained the transcriptome data of callose GSL genes at different floral developmental stages in *Arabidopsis* 38. The expression levels of the callose GSL and endo‐1,3‐β‐glucosidase genes were calculated using values of fragments per kilobase of exon model per million mapped reads in the root, stem, leaf, and flower in *B. rapa*. Finally, the expression data were normalized gene‐wise and hierarchically clustered based on Pearson's coefficient with average linkage in the genesis (version 1.7.6) program 39.

### RNA extraction and quantitative real‐time PCR

Total RNA was extracted from Chinese cabbage flowers at four developmental stages using TRIzol reagent (Invitrogen, Carlsbad, CA, USA) following the manufacturer's instructions. RNA quality was determined with a NanoDrop ND1000 spectrophotometer (NanoDrop Technologies, Wilmington, DE, USA). A total of 2 μg of total RNA per sample was reverse‐transcribed using oligo (dT) and Superscript II reverse transcriptase (Invitrogen, Thermo Fisher Scientific, Waltham, MA, USA). All of the primers (Tables S1 and S2) were designed by primer‐blast (http://www.ncbi.nlm.nih.gov/tools/primer-blast/index.cgi?LINK_LOC=BlastHome), using the following parameters: 150–200 bp PCR product size, Nr database, 58–62 °C primer melting temperature, and *B. rapa* as the organism (taxid: 3711). All PCR reactions were performed under the following conditions: 40 cycles of 5 s at 94 °C, 15 s at 60 °C, and 34 s at 72 °C. Using FastStart Universal SYBR Green Master (Rox; Roche, Indianapolis, IN, USA) and a 7500 Sequence Detection System (Thermo Fisher Scientific), quantitative real‐time PCR was conducted in triplicate with different cDNAs synthesized from three biological replicates of different tissues and development stages. For each analysis, a linear standard curve, the threshold cycle number versus log (designated transcript level) was constructed using a serial dilution of a specific cDNA standard. The levels of the transcript in all unknown samples were determined according to the standard curve. *Brassica rapa* tubulin β‐2 chain‐like (LOC103873913) was used as an internal standard, and *t*‐test statistical analysis was performed using the software ibm spss statistics 20.0 (IBM Corp., Hong Kong, China) 40.

## Results

### Phenotypic characterization of fertile and sterile floral buds

**Figure 1:**
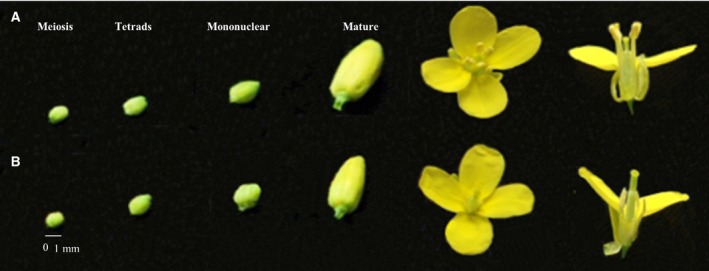
Phenotypic characterization of fertile and sterile flower buds of Chinese cabbage line ‘10L03’ at different developmental stages. (A) Phenotype of fertile buds; (B) phenotype of sterile buds. The four development stages from left to right are meiosis, tetrad, mononuclear and mature. Fertile bud sizes from left to right are 1.10, 1.30, 1.60, and 2.70 mm; sterile bud sizes from left to right are 1.00, 1.20, 1.40, and 2.20 mm.

Different developmental stages (meiosis, tetrad, mononuclear and mature stages) of floral buds are shown in Fig. 1. Sterile flowers were visually smaller than fertile flowers, and no pollen grains were observed in the sterile flower buds (Fig. 1). During the developmental process, the anthers and filaments of the sterile flowers remained shorter than those of the fertile flowers.

**Figure 2:**
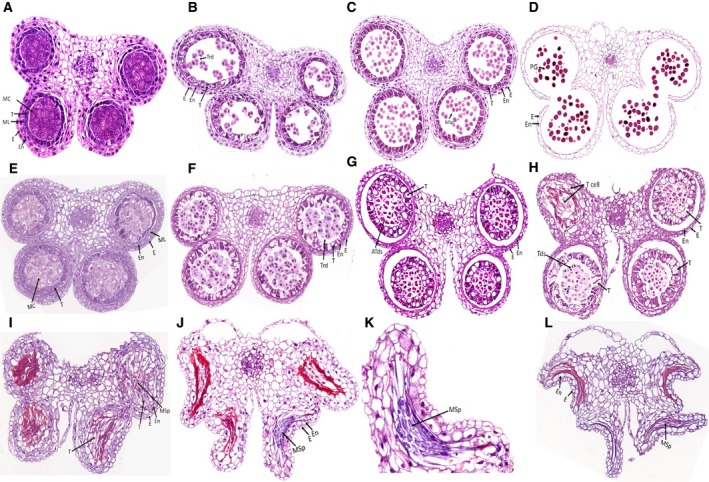
Transverse sections of fertile anthers (A–D) and sterile anthers (E–L) from Chinese cabbage line ‘10L03’. E, epidermis; En, endothecium; MC, meiotic cell; ML, middle layer; MSp, microspores; T, tapetum; Tds, tetrads; PG, pollen grains. (A–D) Development stages 6, 7, 8 and 13 of fertile anthers showed normal pollen development. (E, F) Development stages 6 and 7 of sterile anthers showed similarity to fertile anthers but there was vacuolization in tapetal cells at stage 7. (G–K) Aborted anther in which callose surrounding tetrads could not degrade in time and tetrads could not release microspores. (G) Greater vacuolization in tapetal cell; some tetrads were transformed and abortion occurred. (H, I) Different abortion speeds in one anther. Some tapetal cells enlarged and occupied locules and some degraded. Tetrads could not develop into mononuclear microspores. (J) Aborted anthers in which tapetum structure disappeared. (K) Aborted microspores in the abort anthers. (L) Complete abortion and shrinkage of sterile anthers.

An investigation using paraffin sections of fertile and sterile anthers is shown in Fig. 2 30. Microspores developed normally (Fig. 2A–C, stage 6, 7, 8) and numerous mature pollen grains could be observed (Fig. 2D, stage 13) in fertile anthers. Compared with fertile anthers (Fig. 2A,B), in sterile anthers there were no obvious differences in the cellular morphology of the epidermis, endothecium, middle layer, and microsporocytes at stage 6 (Fig. 2E), and at stage 7 normal tetrads could be formed (Fig. 2F), but we could observe vacuolization in tapetal cells of sterile anthers at stage 7 (Fig. 2F). Sterile tetrads were malformed and abortion occurred while the vacuolization of tapetal cells was increased (Fig. 2G) compared with stage 7. The pollen sac abortion was different in one anther (Fig. 2H,I). Some tapetal cells enlarged and occupied locules and some degraded (Fig. 2). Some tetrads seemed normal, others were abortive (Fig. 2H). Tetrads could not develop into mononuclear microspores (Fig. 2) and some became abortive microspores without viability (Fig. 2K) while the tapetum structure finally disappeared (Fig. 2J). At the late pollen developmental stage, sterile anther locules had sequentially shrunk and shriveled (Fig. 2L). We could not observe any microspores released from tetrads (Fig. 2G–L), and the possible reason for microspore abortion was that callose surrounding tetrads could not degrade in time and tetrads could not develop into mononuclear microspores further.

### Abnormal callose deposition during the abortion of microspores in Chinese cabbage

**Figure 3:**
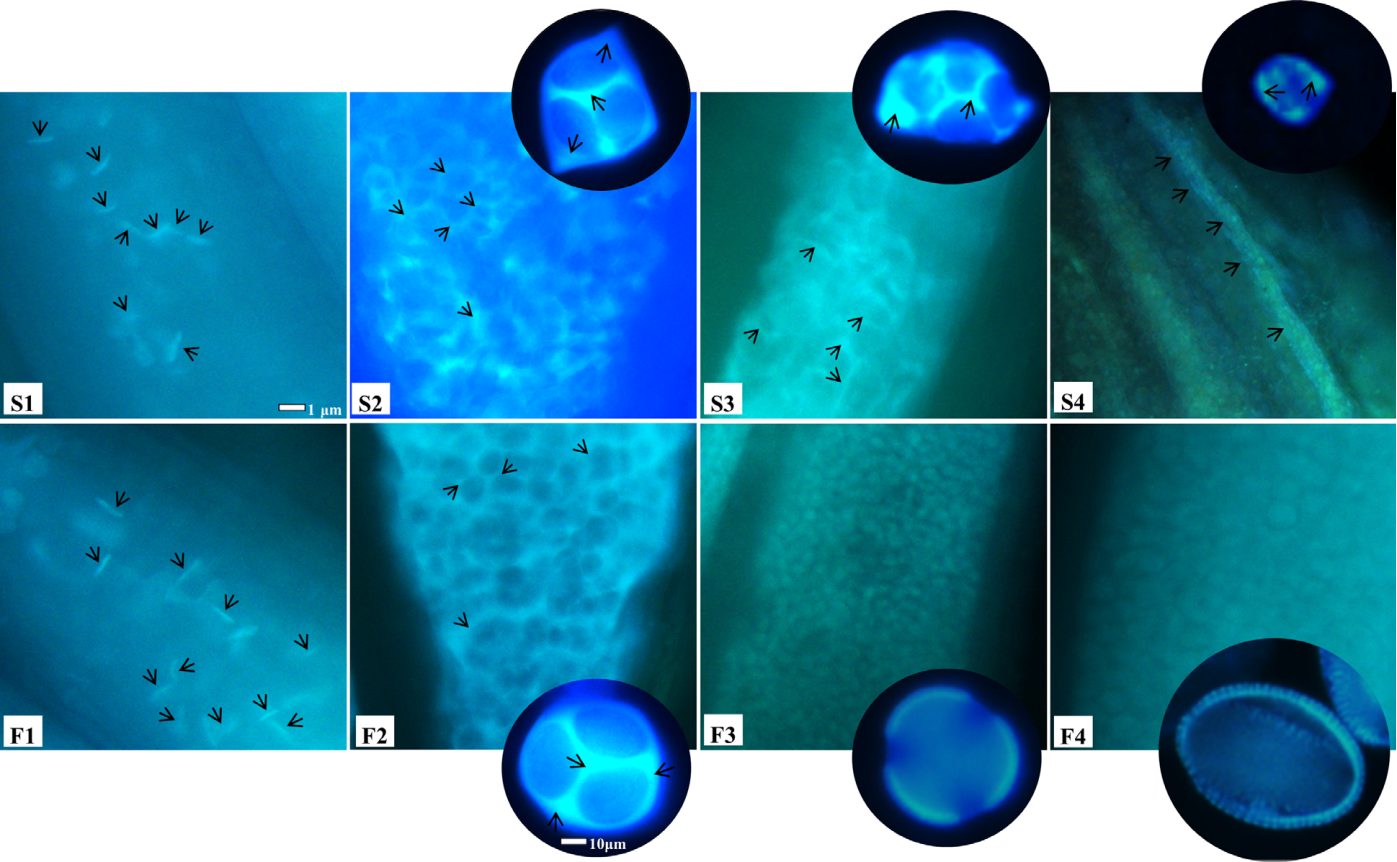
Callose deposition of fertile and sterile anthers from Chinese cabbage line ‘10L03’. F1–F4 show callose deposition of fertile anthers at meiosis, tetrad, mononuclear and mature stages with aniline blue staining. S1–S4 show callose deposition of sterile anthers at those developmental stages corresponding to fertile anthers. The arrows indicate the fluorescence signal emitted by callose. Scale bar: 1 μm.

To test our hypothesis, callose deposition was examined during microspore development in fertile and sterile anthers. Results are shown in Fig. 3, where panels F1–F4 show normal fluorescence signals emitted by callose at meiosis, tetrad, mononuclear and mature pollen stages in fertile microspores. Some short linear fluorescence emitted by pollen mother cells could be observed at the meiosis stage from fertile anthers (Fig. 3, F1). Fluorescence signals were the strongest at tetrad stage, and appeared on the surface and the template of tetrads (Fig. 3, F2). Then, the callose degraded, the fluorescence signals on the surface and the template of tetrads disappeared, and the tetrads released mononuclear microspores. After the tetrads had released mononuclear microspores, callose accumulated on the pollen exine and fluorescence signals were detected there (Fig. 3, F3 and F4). Compared with fertile anthers, sterile anthers showed similar callose deposition to fertile anthers at meiosis and tetrad stage, but the tetrads were malformed (Fig. 3, F1 and S1; F2 and S2). However, the fluorescence response was stronger in sterile than in fertile anthers at mononuclear stages (Fig. 3, F3 and S3) and was expressed similar to the tetrads stage (Fig. 3, S2 and S3), indicating that the callose on the surface and the template of tetrads in sterile a anther could not be degraded in time. Microspores were not released and gradually broke down, and no mature pollen was produced (Fig. 3, S4). Until anther complete abortion, we could still detect fluorescence on aborted microspores (Fig. 3, S4), indicating that callose accumulation accompanied microspore abortion.

### Identification of the callose GSL and endo‐1,3‐β‐glucosidase enzyme families in Chinese cabbage

To determine which gene was responsible for the abnormal degradation of callose, a genome‐wide analysis of the GSL and endo‐1,3‐β‐glucosidase enzyme families was performed in Chinese cabbage. We used 12 GSL and 25 endo‐1,3‐β‐glucosidase coding sequences from *Arabidopsis* as queries to search against the published genome of Chinese cabbage. After the search, we identified 15 GSLs and 77 endo‐1,3‐β‐glucosidases (Tables 1 and 2). The full‐length GSL proteins in Chinese cabbage ranged from 1768 (*BraA09g025290*) to 2290 (*BraA07g023720*) amino acids and shared a high level of similarity to the *Arabidopsis thaliana* proteins. In terms of biochemical properties, all of the GSL proteins were alkaline, with pI values ranging from 8.55 (*BraA05g008500*) to 9.24 (*BraA05g012120*). The polypeptides were also predicted to contain 14–20 TMHs. Furthermore, we predicted the probable protein localization for each of the candidate callose GSL and endo‐1,3‐β‐glucosidase enzymes in Chinese cabbage using the protein subcellular localization prediction software wolf psort (http://wolfpsort.org). All candidate callose GSL enzymes were most likely localized in the plasma membrane (Table 1). The endo‐1,3‐β‐glucosidases identified in our study had isoelectric points (pI) ranging from 4.61 (*BraA07g008730*) to 9.73 (*BraA07g022070*), with coding sequences of 172–962 amino acids (Table 2). These ranges suggested the biochemical properties of the endo‐1,3‐β‐glucosidase enzyme family in Chinese cabbage were diverse. All candidate callose endo‐1,3‐β‐glucosidase enzymes were most likely localized in the chloroplast and plasma membrane (Table 2).

**Table 1:** Summary information of callose GSL enzymes in Chinese cabbage databases. TMHs were predicted with tmhmm server. psort predictions: C, cytosol; Ch, chloroplast; E, extracellular matrix; ER, endoplasmic reticulum; G, Golgi apparatus; M, mitochondrion; N, nuclear; P, plasma membrane; V, vacuolar membrane.

Gene name	*Arabidopsis thaliana* callose GSL enzymes family	Genomic position	Protein length (Bra/AT)	pI	GRAVY	No. of TMHs	psort predictions
*BraA10g004390*	ATGSL1	A10: 2304458 … 2314452 (−)	1960/1950	9.17	−0.067	14	P: 10, Ch: 2, M: 1
*BraA05g012120*	ATGSL2	A05: 6560336 … 6570308 (−)	1981/1950	9.24	−0.072	15	P: 11, M: 1, E.R.: 1
*BraA02g004600*	ATGSL3	A02: 2211008 … 2220782 (−)	1953/1955	9.08	−0.116	15	P: 10.5, C_P: 6.5, C: 1.5
*BraA10g025720*	ATGSL3	A10: 17256935 … 17266929 (+)	2008/1955	9.00	−0.080	17	C:7, N:3, P:3
*BraA09g010050*	ATGSL5	A09: 5714522 … 5724771 (+)	1928/1923	9.08	0.028	11	P: 5, Ch: 4, C: 2, N: 3
*BraA07g023720*	ATGSL6	A07: 18803988 … 18817761 (+)	2290/1921	9.06	−0.050	20	P: 12, E.R.: 2
*BraA09g063900*	ATGSL7	A09: 43987002 … 43997996 (−)	2063/1958	8.61	−0.210	15	P: 12, N: 1
*BraA10g005220*	ATGSL7	A10: 2779232 … 2789744 (+)	1921/1958	8.78	−0.171	14	P: 13
*BraA05g032510*	ATGSL8	A05: 23552881 … 23568209 (−)	1974/1976	8.70	−0.119	14	P: 10, E.R.: 2, M: 1
*BraA03g033140*	ATGSL9	A03: 16436892 … 16424818 (−)	1938/1890	8.96	0.001	14	P: 12, E.R.: 1
*BraA05g038460*	ATGSL9	A05: 26274883 … 26288188 (+)	1966/1890	8.70	−0.031	15	P: 12, E.R.: 2
*BraA01g041620*	ATGSL9	A01: 27864815 … 27876283 (+)	1895/1890	8.65	−0.015	14	P: 11.5, C_P: 6.5, E.R.: 2
*BraA05g008500*	ATGSL10	A05: 4419011 … 4432819 (+)	1911/1904	8.55	0.023	16	P: 10, M: 2, E.R.: 1
*BraA09g025290*	ATGSL11	A09: 16984666 … 16990065 (−)	1768/1768	9.21	0.068	17	P: 13
*BraA09g002650*	ATGSL12	A09: 1670703 … 1676327 (+)	1782/1780	9.13	−0.025	12	P: 13

**Table 2:** Summary information of callose endo‐1,3‐β‐glucosidase enzymes in Chinese cabbage databases. TMHs predicted with tmhmm Server. psort predictions: C, cytosol; Ch, chloroplast; E, extracellular matrix; ER; endoplasmic reticulum; G, Golgi apparatus; M, mitochondrion; N, nuclear; P, plasma membrane; V, vacuolar membrane.

Gene name	*Arabidopsis thaliana* callose GSL enzymes family	Genomic position	protein length (bra/at)	pI	GRAVY	No. of TMHs	psort predictions
*BraA04g003430*	AT3G57260	A04: 2147011 … 2148136 (+)	344/339	5.19	−0.298	1	E: 5, E.R.: 3, C: 2, N: 1, M: 1, V: 1
*BraA07g038740*	AT3G57260	A07: 26598727 … 26599695 (+)	322/339	6.76	−0.302	0	Ch: 7, M: 4, N: 2
*BraA04g003400*	AT3G57260	A04: 2124561 … 2125079 (+)	172/339	5.45	−0.190	0	Ch: 6, N: 3, C: 3, V: 1
*BraA04g003410*	AT3G57260	A04: 2136295 … 2137413 (+)	340/339	5.16	−0.308	0	Ch: 13
*BraA04g003380*	AT3G57260	A04: 2108150 … 2109289 (+)	347/339	8.45	−0.406	0	Ch: 4,E:3, V: 2, M: 2, N: 1, E.R.:1
*BraA04g003370*	AT3G57260	A04: 2092596 … 2093717 (+)	346/339	4.98	−0.285	1	E: 6, E.R.: 3, Ch: 2, M: 2
*BraA09g047880*	AT3G57270	A09: 36132129 … 36133169 (−)	343/340	8.58	−0.483	0	Ch: 10.5, Ch_M: 6.5, C: 2
*BraA09g047910*	AT3G57270	A09: 36146800 … 36147890 (−)	329/340	4.70	−0.244	0	Ch: 5, M: 2, E.R._P: 2, P: 1.5, E.R.: 1.5, N: 1, C: 1, E: 1
*BraA09g047900*	AT3G57270	A09: 36136273 … 36136926 (−)	217/340	4.87	−0.399	0	Ch: 4, C: 3, M: 3, N: 2.5, cysk_N: 2
*BraA07g022770*	AT3G57240	A07:18282623 … 18283736 (−)	339/341	9.13	−0.162	1	Ch: 13
*BraA04g003390*	AT3G57240	A04: 2115881 … 2117955 (+)	296/341	9.51	−0.229	0	Ch: 6.5, Ch_M: 5, M: 2.5, P: 2, N: 1, C: 1
*BraA02g013450*	AT3G57240	A02: 6780677 … 6782563 (−)	460/341	5.33	−0.170	0	Ch: 6, E: 3, V: 2, N: 1, M: 1
*BraA04g003420*	AT3G57240	A04: 2142311 … 2142998 (+)	189/341	7.00	−0.174	1	Ch: 8, E: 4, C: 1
*BraA01g020550*	AT4G16260	A01: 11055747 … 11057545 (+)	357/344	6.63	−0.157	0	Ch: 4, M: 3, V: 2.5, E.R._V: 2.5, E: 2, E.R.: 1.5
*BraA10g016350*	AT5G58480	A10: 12626253 … 12627804 (−)	478/476	8.34	−0.484	0	P: 8, V: 3, E.R.: 2
*BraA01g019430*	AT1G64760	A01: 10382610 … 10383971 (−)	411/481	5.72	−0.072	0	C: 7, N: 2, Ch: 2, E: 2
*BraA10g021890*	AT1G64760	A10: 15573107 … 15574709 (+)	492/481	5.16	−0.146	1	G_P: 5, P: 4.5, G: 4.5, V: 3, E.R.: 2
*BraA05g042280*	AT1G64760	A05: 28274179 … 28276590 (−)	492/481	5.04	−0.104	1	Ch: 4, P: 4, E.R.: 3, G: 2
*BraA09g011880*	AT1G64760	A09: 7003829 … 7005548 (+)	477/481	4.84	−0.008	0	P: 7, V: 3, E.R.: 2,G: 2
*BraA09g008430*	AT1G64760	A09: 4824792 … 4827239 (+)	485/481	5.69	−0.064	2	P: 11, V: 1, E.R.: 1
*BraA02g044780*	AT1G64760	A02: 30805693 … 30808003 (+)	485/481	6.80	−0.177	2	Ch: 4, V: 3, E.R.: 2, G: 2, N: 1, M: 1
*BraA07g008730*	AT1G64760	A07: 8723989 … 8725473 (−)	494/481	4.61	−0.065	0	Ch: 4, V: 4, G: 3, E: 2
*BraA03g009860*	AT4G31140	A03: 4238534 … 4240122 (−)	505/484	8.31	−0.116	0	Ch: 10, P: 2, N: 1
*BraA02g009170*	AT4G31140	A02: 4324568 … 4326190 (−)	496/484	7.99	−0.117	0	Ch: 9, E: 2, pero: 2
*BraA01g006680*	AT4G31140	A01: 3190104 … 3192089 (−)	483/484	6.10	0.041	1	P: 10, V: 3
*BraA03g056410*	AT4G31140	A03: 29607481 … 29609667 (+)	487/484	5.55	0.075	1	P: 11, V: 3
*BraA02g011800*	At5G58090	A02: 5720115 … 5722306 (+)	478/477	5.66	−0.036	1	C: 7.5, C_N: 4.5, Ch: 4, P: 2
*BraA10g016100*	At5G58090	A10: 12523116 … 12525242 (−)	480/477	6.01	0.001	1	P: 7.5, G_P: 6, G: 3.5, V: 2
*BraA02g024430*	AT5G20560	A02: 14473651 … 14474938 (−)	348/337	9.44	−0.161	1	Ch: 12, M: 1
*BraA02g008950*	AT5G20560	A02: 4216542 … 4217564 (+)	340/337	4.78	0.089	1	Ch: 7, C: 3, E.R.: 2, E: 1
*BraA01g030220*	AT5G20560	A01: 20262807 … 20264430 (−)	476/337	7.57	−0.143	0	Ch: 12, N: 1
*BraA10g020080*	AT5G20560	A10: 14635926 … 14636972 (−)	348/337	5.54	0.064	0	C: 11, Ch: 2
*BraA02g008840*	AT5G20330	A02: 4163389 … 4164420 (+)	343/345	6.52	0.069	1	V: 4.5, Ch: 3, E.R._V: 3, M: 2, N: 1, C: 1, E: 1
*BraA05g038340*	AT3G07320	A05: 26227570 … 26229136 (+)	460/460	8.76	−0.139	0	V: 5, Ch: 3, G: 3, N: 1, C: 1
*BraA03g033200*	AT3G07320	A03: 16455438 … 16455438 (−)	566/460	7.07	−0.080	0	V: 5, Ch: 4, G: 2, N: 1, C: 1
*BraA05g006430*	AT3G07320	A05: 3261622 … 3263594 (+)	448/460	5.34	−0.141	0	E: 8, Ch: 3, C: 1, M: 1
*BraA07g022070*	AT3G07320	A07: 17863644 … 17865532 (−)	449/460	9.73	−0.051	1	E: 4, Ch: 2, V: 2, E.R.: 2, G: 2, M: 1
*BraA04g004630*	AT3G07320	A04: 2952135 … 2954025 (+)	451/460	9.59	−0.144	1	E: 8, G: 2, Ch: 1, M: 1, V: 1
*BraA09g046240*	AT3G07320	A09: 35175900 … 35177882 (−)	453/460	9.05	−0.069	1	E: 5, V: 3, G: 2, Ch: 1, N: 1, M: 1
*BraA09g046250*	AT3G07320	A09: 35184582 … 35186606 (−)	450/460	8.78	−0.120	1	E: 10, Ch: 1, C: 1, M: 1
*BraA10g020220*	AT4G14080	A10: 14699022 … 14700059 (−)	345/478	4.81	0.106	1	V: 3, Ch: 2, M: 2, E: 2, E.R.: 2, N: 1, C: 1
*BraA07g009320*	AT4G14080	A07: 9280417 … 9282035 (−)	474/478	6.24	−0.137	0	Ch: 11, N: 2
*BraA03g040850*	AT4G14080	A03: 20384316 … 20385972 (+)	480/478	7.03	−0.114	1	Ch: 12, N: 1
*BraA04g008040*	AT4G14080	A04: 5587773 … 5589425 (+)	480/478	8.64	−0.178	0	Ch: 10, E: 3
*BraA08g009700*	AT4G14080	A08: 8598442 … 8600053 (−)	505/478	8.92	−0.218	0	Ch: 11, N: 2
*BraA05g015330*	AT4G14080	A05: 8949349 … 8951030 (−)	491/478	8.45	−0.174	1	Ch: 10, N: 1, E: 2
*BraA01g008280*	AT4G29360	A01: 3995994 … 3997972 (+)	532/534	4.99	−0.023	1	P: 7, G: 3, E.R.: 2, E: 1
*BraA08g018600*	AT4G29360	A08: 14662588 … 14664824 (+)	520/534	4.82	−0.051	0	Ch: 7, V: 3, E.R.: 2, N: 1
*BraA10g014510*	AT5G56590	A10: 11663337 … 11664988 (+)	492/506	6.93	−0.256	0	N: 4, Ch: 3, C: 2, P: 2, G: 2
*BraA04g003450*	AT4G34480	A04: 2156824 … 2157667 (+)	252/504	8.38	−0.172	0	Ch: 9, V: 3.5, E.R. _V: 2.5
*BraA10g013180*	AT4G34480	A10: 10798213 … 10800027 (+)	468/504	5.09	0.047	0	Ch: 6, M:2, E:2,V:2, N:1
*BraA03g013540*	AT4G34480	A03: 5909250 … 5910863 (−)	460/504	5.84	−0.137	0	Ch: 4, E: 3, M: 2, V: 2, E.R.: 2
*BraA07g007140*	AT4G34480	A07: 7404663 … 7406549 (−)	473/504	6.47	−0.190	0	Ch: 4, V: 4, E.R.: 2, N: 1, M: 1, E: 1
*BraA09g010430*	AT4G34480	A09: 6005312 … 6008369 (−)	512/504	4.81	−0.019	1	P: 8, V: 4, Ch: 1
*BraA01g003770*	AT4G34480	A01: 1855992 … 1864107 (+)	962/504	6.74	−0.200	1	P:7, V:3, G:2, N:1
*BraA03g058860*	AT4G34480	A03: 31176827 … 31179475 (−)	456/504	5.52	−0.092	0	P:1. 5, V: 2, E: 6, Ch: 1, E.R.: 2.5
*BraA06g044140*	AT4G34480	A06: 28721027 … 28723694 (−)	432/504	5.90	−0.050	0	Ch: 8, P: 2, N: 1, E: 1, E.R.: 1
*BraA09g006190*	AT4G34480	A09: 3603345 … 3605020 (+)	449/504	5.95	−0.055	1	CYSK:7, Ch: 5, CYTO:2
*BraA01g036400*	AT2G27500	A01: 24818100 … 24820228 (−)	391/392	9.09	−0.062	1	P: 9.5, C_P: 5.5, E: 2, Ch: 1
*BraA05g031250*	AT2G27500	A05: 22911521 … 22913884 (−)	359/392	9.47	−0.273	1	C: 6, Ch: 2, M: 2, E.R.: 2, N: 1
*BraA03g025370*	AT2G27500	A03: 12501173 … 12502355 (−)	365/392	7.63	−0.092	0	Ch: 5, C: 2, M: 2, N: 1, E: 1, V: 1, E.R.: 1
*BraA03g048190*	AT2G27500	A03: 24399262 … 24401238 (−)	397/392	7.77	−0.055	1	Ch: 11, V: 3
*BraA08g012890*	AT2G27500	A08: 11094916 … 11096686 (−)	379/392	5.98	−0.049	1	Ch: 5, E: 4, V: 3, E.R.: 2
*BraA01g009790*	AT2G27500	A01: 4981541 … 4985567 (−)	715/392	6.61	−0.087	1	Ch: 6, V: 4, N: 1, E:1
*BraA09g034950*	AT2G27500	A09: 27672620 … 27673920 (+)	378/392	5.77	−0.035	0	V: 4, Ch: 4, M:2, E:2, N:1
*BraA07g018180*	AT2G27500	A07: 15530481 … 15531596 (−)	371/392	6.97	0.054	0	Ch: 7, N:2, G:2, P:1, E: 1
*BraA03g025090*	AT2G27500	A03: 12365391 … 12367361 (+)	390/392	7.82	−0.027	0	Ch: 10, N: 2, M: 2
*BraA09g032320*	AT1G32860	A09: 25117099 … 25118740 (+)	422/426	6.61	−0.004	1	Ch:6,P: 2, V: 2, N: 1, C: 1, M: 1
*BraA07g020840*	AT5G42100	A05: 17223875 … 17225167 (−)	430/425	7.60	−0.000	1	Ch: 3, V: 3, M: 2, E: 2, G: 2, P: 1
*BraA09g046540*	AT1G11820	A09: 35413767 … 35414900 (+)	377/511	7.02	0.316	1	Ch: 9, M: 2, V: 2
*BraA07g022860*	AT1G11820	A07: 18329183 … 18330492 (+)	408/511	7.08	0.231	2	P: 7, V: 3, E.R.: 2, M: 1
*BraA02g045500*	AT1G11820	A02: 31218872 … 31220166 (+)	379/511	7.59	−0.194	0	V: 6, Ch: 3, E: 3, C: 1
*BraA07g024880*	AT1G11820	A07: 19368262 … 19369631 (+)	366/511	9.77	−0.012	0	Ch: 10, N: 1, C: 1, M: 1
*BraA08g031380*	AT1G11820	A08: 21054111 … 21055943 (+)	519/511	5.62	0.034	0	P: 8, V: 2, Ch: 1,C: 1, E.R.: 1
*BraA05g033550*	AT3G13560	A05: 24116562 … 24118289 (+)	501/505	5.09	0.022	0	P: 9, V: 3, E.R.: 1
*BraA02g016980*	AT1G66250	A02: 9131279 … 9132971 (+)	468/505	5.57	−0.028	0	Ch: 14
*BraA02g034940*	AT2G01630	A02: 24164042 … 24166367 (−)	497/501	5.23	0.106	0	P: 8, V: 3, Ch: 1, E.R.: 1

### Phylogenetic and gene structural analyses of the callose GSL and endo‐1,3‐β‐glucosidase enzymes in Chinese cabbage

**Figure 4:**
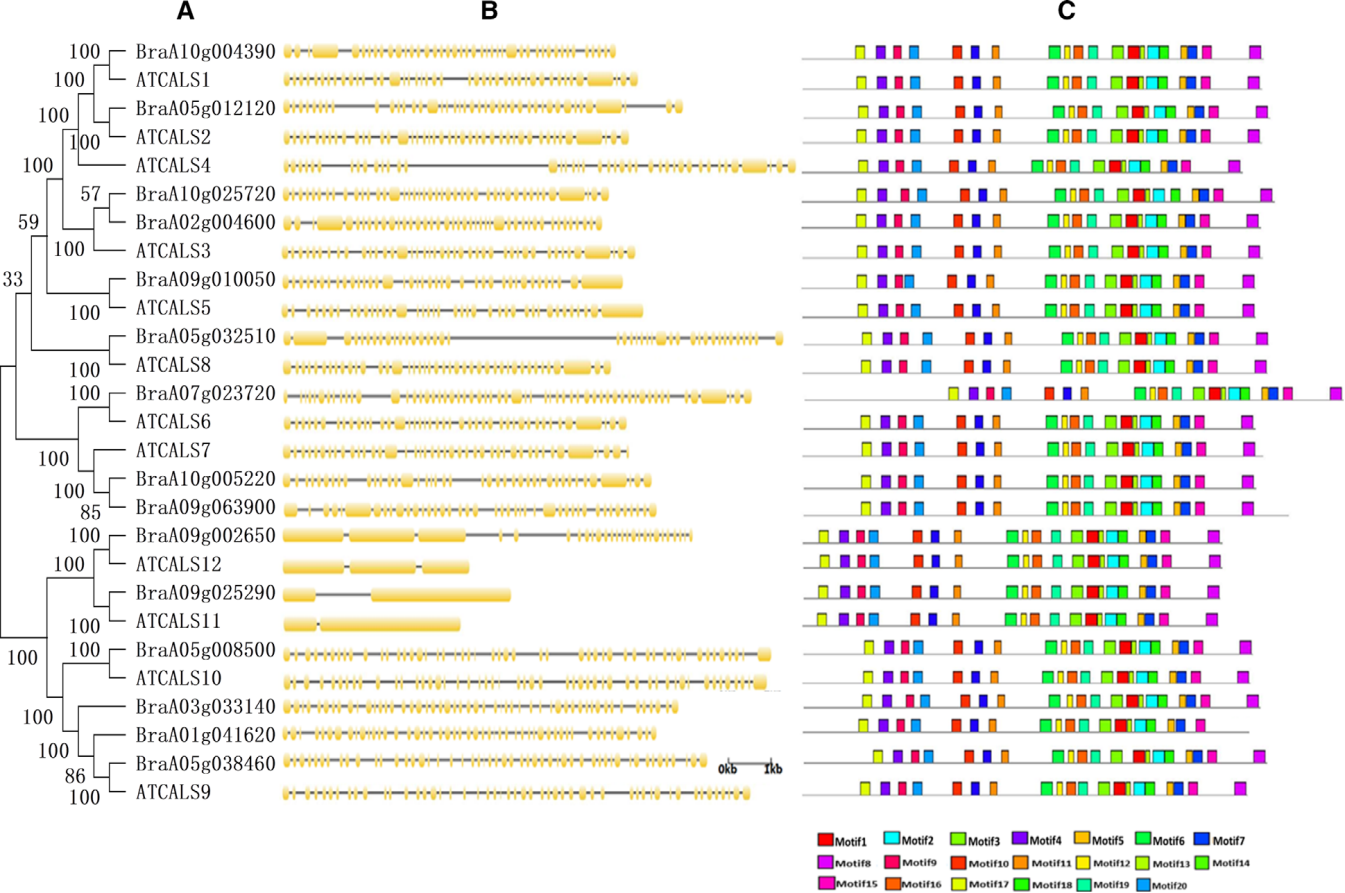
Phylogenetic relationships and gene structures among 15 Chinese cabbage callose GSL proteins. (A) Phylogenetic relationships. (B) exon–intron structures. (C) conserved motifs. The molecular phylogeny (left panel) was constructed using full‐length callose GSL protein sequences from Chinese cabbage. Numbers associated with branches show bootstrap support values for maximum‐likelihood analyses and posterior probabilities for Bayesian analyses, respectively. The 20 major groups, designated from 1 to 20, are marked with different colored backgrounds. Exon–intron structures of the callose GSL genes are shown in the middle panel. Yellow boxes represent exons and black lines represent introns. A schematic representation of conserved motifs (obtained using meme) in callose GSL proteins is displayed in the panel on the right. Different motifs are represented by different colored boxes. Number of the individual motifs is shown in Table S3.

**Figure 5:**
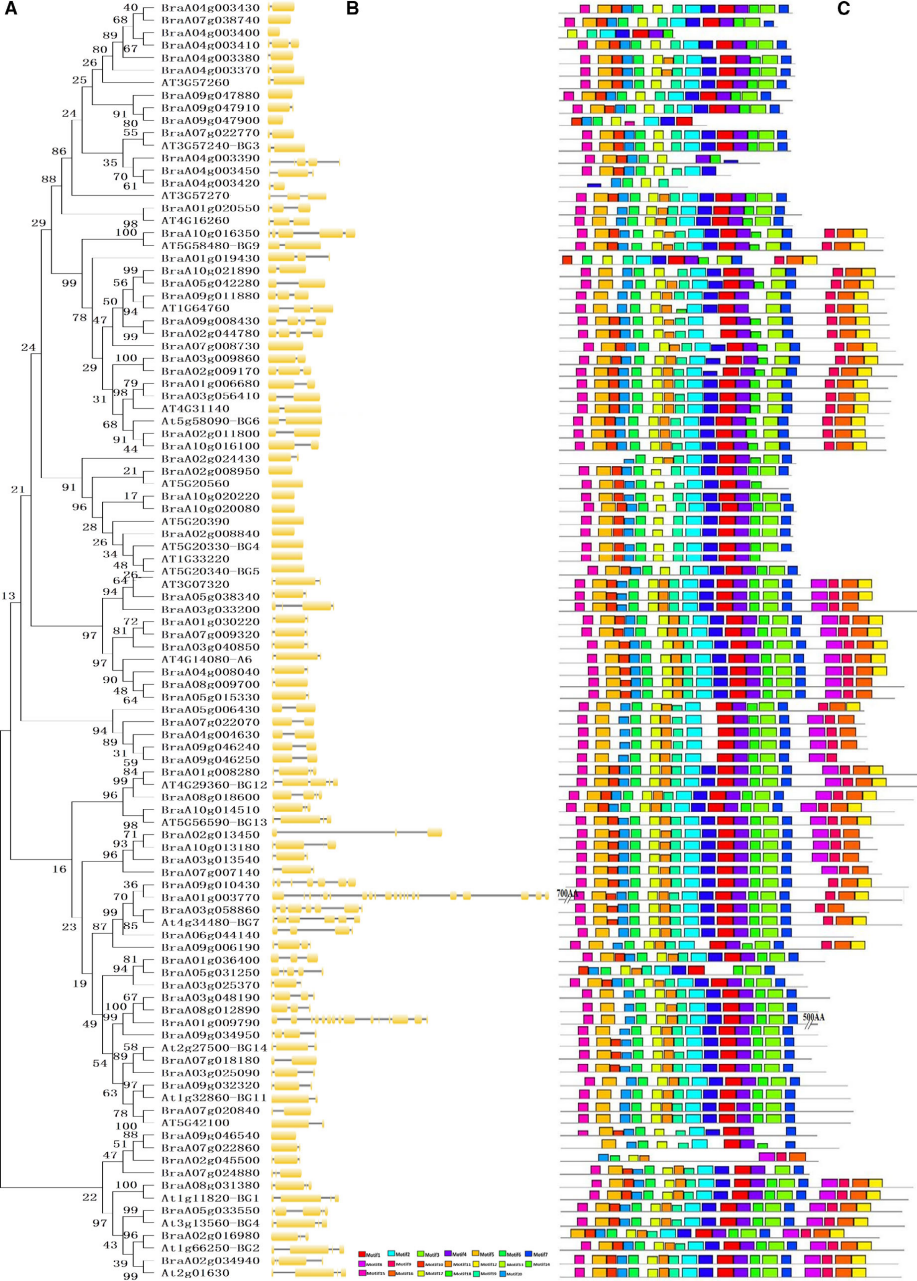
Phylogenetic relationships and gene structures among 77 Chinese cabbage callose endo‐1,3‐β‐glucosidase proteins. (A) Phylogenetic relationships. (B) Exon–intron structures. (C) Conserved motifs. The molecular phylogeny (left panel) was constructed using full‐length callose endo‐1,3‐β‐glucosidase protein sequences from Chinese cabbage. Numbers associated with branches show bootstrap support values for maximum likelihood analyses and posterior probabilities for Bayesian analyses, respectively. The 20 major groups, designated from 1 to 20, are marked with different colored backgrounds. Exon–intron structures of the callose endo‐1,3‐β‐glucosidase genes are shown in the middle panel. Yellow boxes represent exons and black lines represent introns. A schematic representation of conserved motifs (obtained using meme) in callose endo‐1,3‐β‐glucosidase proteins is displayed in the panel on the right. Different motifs are represented by different colored boxes. Number of the individual motifs is shown in Table S4.

We generated phylogenetic trees for each family to gain insight into the phylogenetics of both the GSL and endo‐1,3‐β‐glucosidase enzyme families. We also compared the evolutionary relationships between Chinese cabbage and *Arabidopsis* GSLs and endo‐1,3‐β‐glucosidase enzymes (Figs 4 and 5). All GSLs in Chinese cabbage were clustered closely with AtGSLs. In addition, we compared intron–exon numbers, lengths, distribution and other genetic structures to understand the evolutionary history of the gene families 41. The results showed that all the GSLs in Chinese cabbage were intron‐rich, with 24–57 introns (except *BraA09g025290* with one intron), and that the *Arabidopsis* and *B. rapa* callose GSL enzymes had similar coding sequences and very similar exon–intron structures, strongly supporting their close evolutionary relationships (Fig. 4). The loss of *BraA09g025290* introns might have resulted in functional differences. In addition, all members of the callose GSL enzymes possessed 20 motifs (Fig. 4, Table S3). As expected, all of the most closely related members had common motif compositions, suggesting functional similarities among the callose GSL enzyme proteins.

According to the phylogenetic tree of the endo‐1,3‐β‐glucosidase enzymes, the number of exons ranged from 1 to 5. The structural differences in the enzymes might allow them to perform different functions. Gene structure diversification is a direct expression of gene family expansion 41. The structural diversity of callose endo‐1,3‐β‐glucosidase enzyme family members in Chinese cabbage provides a mechanism for gene evolution, and exon loss or gain can be an important step in generating structural diversity and complexity 42. In this study, 13 exons were found in *BraA01g009790* (Fig. 5), and *BraA01g003770* had 20 exons, which indicates that the gain of exons might have resulted in functional divergence. In addition, we searched for conserved motifs within the Chinese cabbage endo‐1,3‐β‐glucosidase enzymes using online meme tools to analyze their motif compositions. A total of 20 conserved motifs, designated motif 1 to motif 20, were identified within the genes. These motifs may help to predict the genes’ functions. As shown in Fig. 5 and Table S4, the number of motifs of the endo‐1,3‐β‐glucosidase enzyme family ranged from 5 to 18, and most had 14. Differences in motif composition may have resulted in functional divergence between different genes. However, it is uncertain whether such differences have led to loss of function and this needs further investigation and molecular experimental verification. Motifs 6 and 15 were shared by all the Chinese cabbage callose endo‐1,3‐β‐glucosidase enzyme members and were relatively conserved. The phylogenetic and structural analyses presented here may facilitate the functional annotation and study of callose GSL and endo‐1,3‐β‐glucosidase enzymes in Chinese cabbage.

### Chromosomal locations of the callose GSL and endo‐1,3‐β‐glucosidase genes in the genome

To investigate the genomic distribution of the predicted callose GSL and endo‐1,3‐β‐glucosidase genes, the DNA sequences were used to search the *B. rapa* genome database. We found that the callose GSL genes were distributed unevenly among the 10 chromosomes of the Chinese cabbage genome (Fig. S1). Four callose GSL genes were found on chromosomes chr5 and chr9, three on chromosome chr10, and one on each on chromosomes chr1, chr2, chr3, and chr7. No callose GSL genes were found on chr4, chr6 or chr8. The callose endo‐1,3‐β‐glucosidase genes were also unevenly distributed on the Chinese cabbage chromosomes (Fig. S1). Chromosomes 9 and 6 contained the maximum and minimum numbers, respectively, of callose endo‐1,3‐β‐glucosidase genes.

### Callose GSL and endo‐1,3‐β‐glucosidase gene expression profiles in different tissues of Chinese cabbage

**Figure 6:**
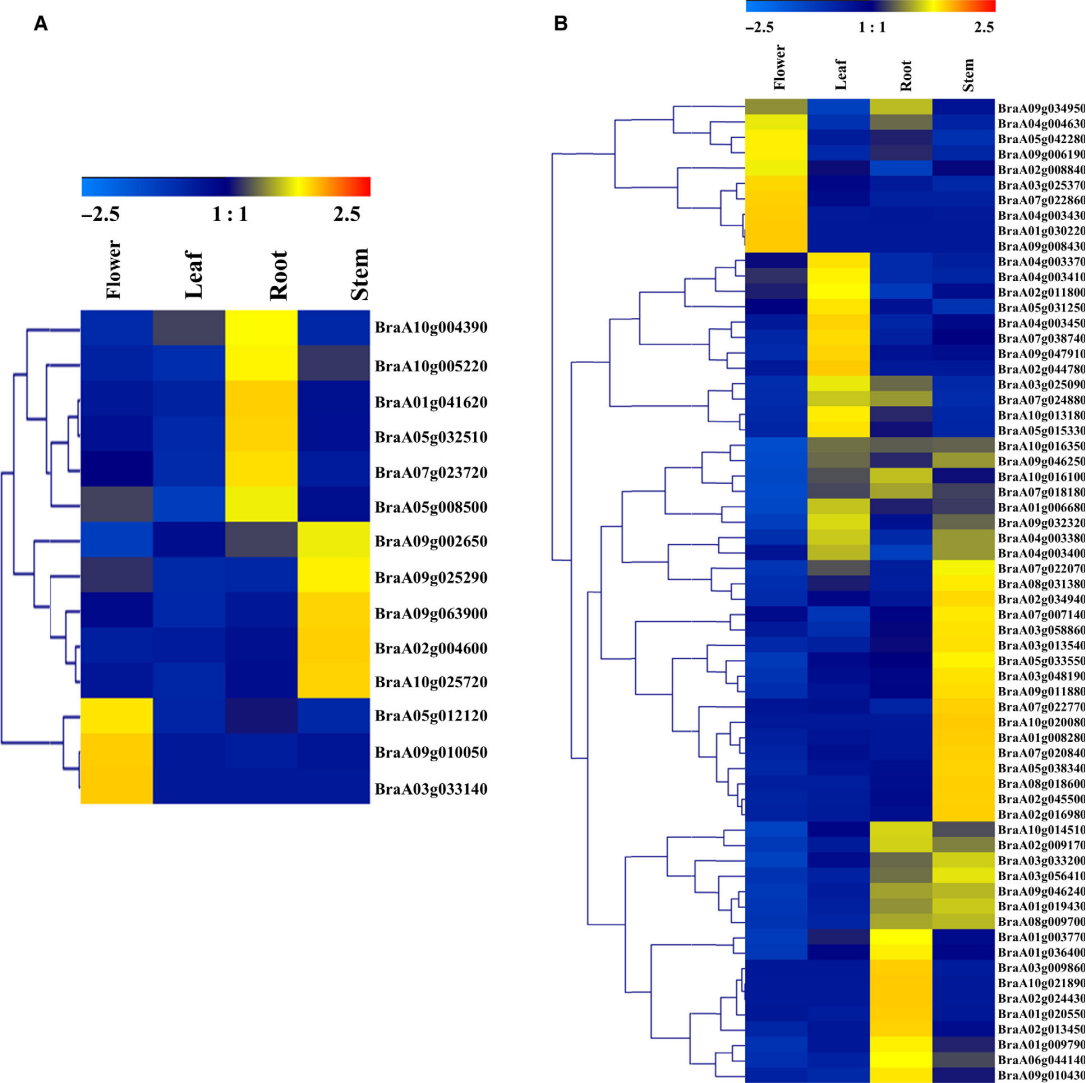
Expression profiles in different tissues of callose GSL and endo‐1,3‐β‐glucosidase genes in Chinese cabbage. (A) Callose GSL genes. (B) Callose endo‐1,3‐β‐glucosidase genes. Dynamic expression profiles were generated using the FPKMs of the callose GSL and endo‐1,3‐β‐glucosidase genes in different tissues. FPKM values (log2 ratios) were normalized gene‐wise and hierarchically clustered using genesis software. Highly and weakly expressed genes are colored red and green, respectively; gray represents an FPKM value of 0.

Expression profiles give useful information on gene functions 43. Here, we reanalyzed the expression levels of callose GSL and endo‐1,3‐β‐glucosidase genes using publicly available RNA sequence data of four different tissues 37. We selected the *B. rapa* accession Chiifu‐401‐42 to investigate the callose GSL and endo‐1,3‐β‐glucosidase genes in the root, stem, leaf and flower. We used hierarchical clustering of the gene expression profiles to create heat maps (Fig. 6A,B). The results showed, in different tissues, 15 GSL genes had different expression patterns (Fig. 6A). In certain tissues, some genes were highly expressed. For example, *BraA09g010050* and *BraA05g012120* showed peak transcript levels in the flower. Similarly, the 77 endo‐1,3‐β‐glucosidase genes in Chinese cabbage were differentially expressed in different tissues (Fig. 6B). In certain developmental stages, some genes were highly and specifically expressed. For example, *BraA04g003430* and *BraA09g008430* were only expressed and showed peak transcript levels in the flower. Therefore, these genes may play roles that cannot be neglected during flower development. In addition, the *BraA03g025370*,* BraA05g031250* and *BraA05g042280* genes showed particularly high levels of expression in the flower. Some genes (including *BraA07g022770*,* BraA08g031380*,* BraA02g034940*,* BraA01g008280* and *BraA07g020840*) were highly transcribed in the stem, whereas *BraA02g011800*,* BraA09g047910* and *BraA04g003450* were highly transcribed in the leaf. Conversely, transcripts of some genes, especially*BraA04g008040* and *BraA07g009320*, were not detected in any of the tissues. The transcriptional patterns indicate that these genes may be involved in organ development and growth. The different patterns also suggest the functional divergence of callose endo‐1,3‐β‐glucosidase enzymes.

### Relative expression analysis of callose GSL and endo‐1,3‐β‐glucosidase genes at different floral developmental stages in Chinese cabbage

**Figure 7:**
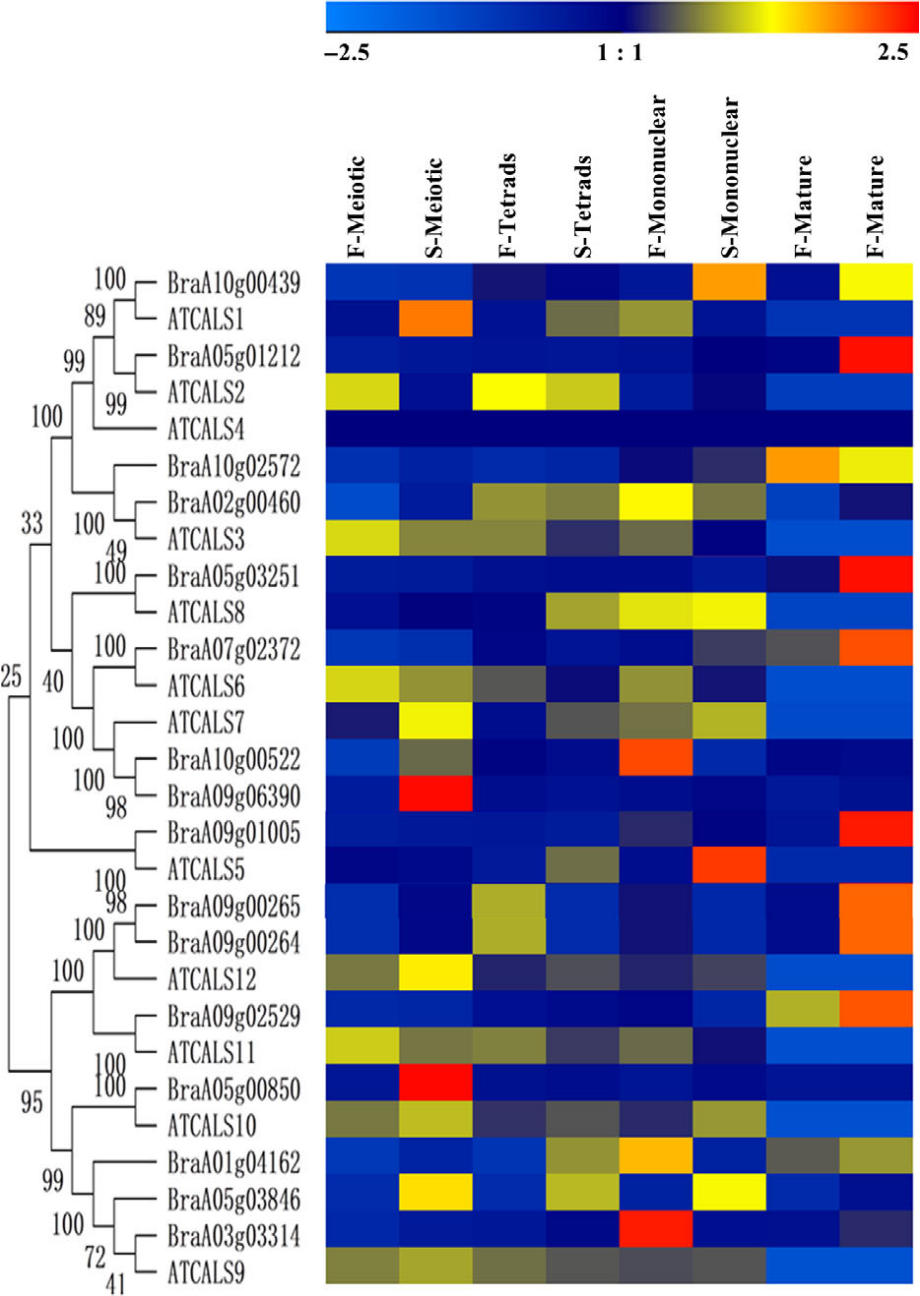
Expression patterns of callose GSL genes at different floral developmental stages in Chinese cabbage and *Arabidopsis*. Relative expression analysis of callose GSL genes in the meiosis, tetrad, mononuclear and mature stages. qPCR analyses were performed, and expression values were calculated using the $2^{-\Delta \Delta C_{T}}$ method. Data are mean values ± SE obtained from three replicates.

**Figure 8:**
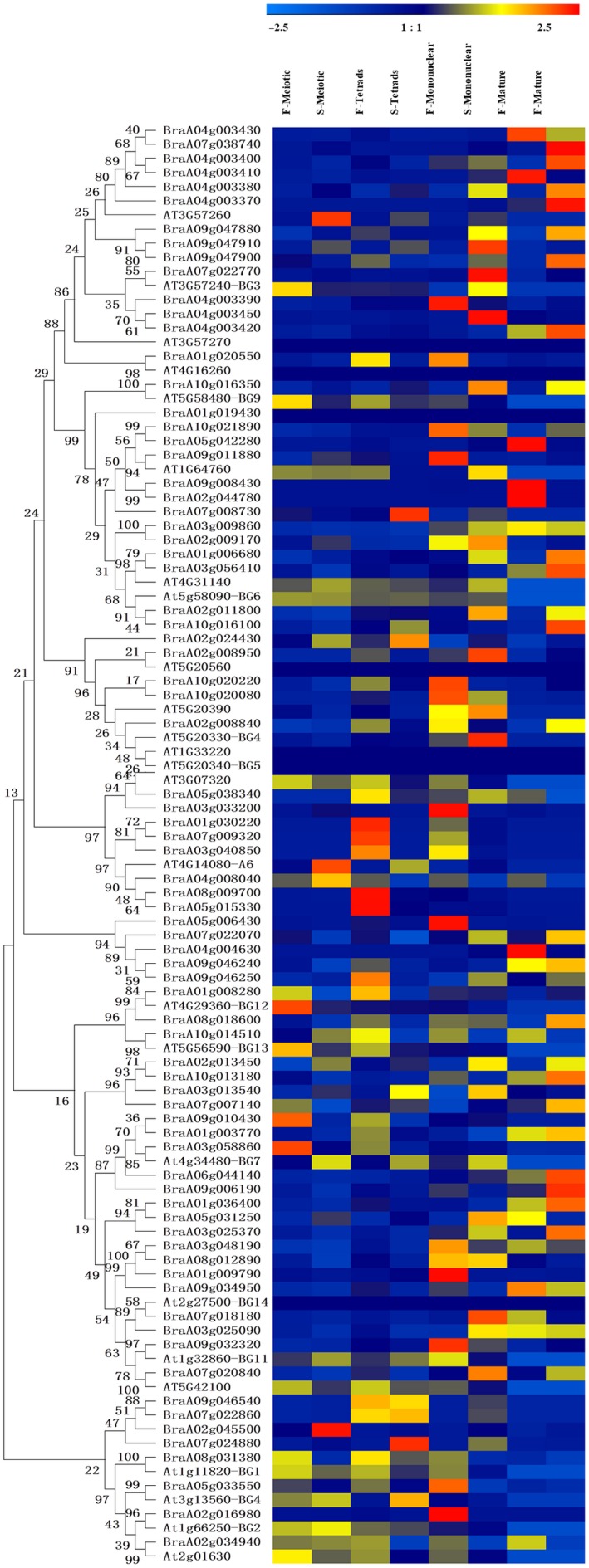
Expression patterns of callose endo‐1,3‐β‐glucosidase genes at different floral developmental stages in Chinese cabbage and *Arabidopsis*. Heat map of qRT‐PCR analysis of callose endo‐1,3‐β‐glucosidase genes in the meiosis, tetrad, mononuclear and mature stages. F, fertile; S, sterile. qPCR analyses were performed, and expression values were calculated using the $2^{-\Delta \Delta C_{T}}$ method. Data are mean values ± SE obtained from three replicates.

Callose is synthesized by GSL and degraded by endo‐1,3‐β‐glucosidase enzymes 1, 44, 45. To determine the reason for the delayed degradation of callose in the tetrad stage, we further investigated the expression profiles of the callose GSL and endo‐1,3‐β‐glucosidase genes in four developmental stages (meiosis, tetrad, mononuclear, and mature) of flowers in Chinese cabbage by conducting real‐time quantitative PCR (qRT‐PCR) analysis. Among the 15 GSL genes assayed in the four flower development stages, *BraA01g041620* showed the most significant increases in the meiosis and tetrad stages in sterile plants (Fig. 7). Among the 77 endo‐1,3‐β‐glucosidase genes, *BraA04g008040*,* BraA07g009320*,* BraA01g030220*,* BraA03g040850*,* BraA10g020080* and *BraA05g038340* exhibited remarkable downregulation in the meiosis and tetrad stages in sterile plants (Fig. 8). These results suggest the reason for the delayed degradation of callose may be a combination of high expression of the synthesis enzyme *BraA01g041620* and low expression of the degradation enzymes *BraA04g008040*,* BraA07g009320*,* BraA01g030220*,* BraA03g040850*,* BraA10g020080* and *BraA05g038340*.

### The expression divergence of callose GSL and endo‐1,3‐β‐glucosidase genes family in *Arabidopsis* and *B. rapa*

To observe clearly the expression divergence between different members in the same gene family, we reanalyzed the expression levels of callose GSL and endo‐1,3‐β‐glucosidase genes using publicly available RNA sequence data of different stages of flower in *Arabidopsis* 38, and our data of the expression of callose GSL and endo‐1,3‐β‐glucosidase genes in fertile and sterile flower buds of the Chinese cabbage were reanalyzed. The results showed callose GSL genes in the same clades have similar expression trends, strongly supporting their close evolutionary relationship (Fig. 4). For example, the GSL genes *AtCAL7*,* BraA10g005220* and *BraA09g063900* are highly expressed during the sterile meiotic stage. But there are still some genes that have undergone functional differentiation (Fig. 4). The genes of the same clades, the *BraA01g041620* and *AtCAL*9 genes, were highly expressed at the sterile meiotic stage, but the *BraA03g033140* and *BraA05g038460* genes were highly expressed in the fertile mononuclear stage. Furthermore, differences were found between clades in the endo‐1,3‐β‐glucosidase enzyme family. In the same clade, *At4G14080* (*At‐A6*) and *BraA04g008040* were detected at a higher transcription level in the sterile meiotic stage; however, the *BraA08g009700* and *BraA05g015330* transcript levels were found to be greater in the fertile tetrad stage.

## Discussion

Pollen development is an extremely important biological process in flowering plants and an indispensable life process for plant genetic breeding 2. Abnormal development of pollen affects its function and can lead to male sterility, which seriously affects the reproduction and evolution of plants 46. In this study, we compared the morphology of fertile and sterile flower buds of cabbage, and found that sterile stamens had no pollen grains when compared with those in fertile flower buds (Fig. 1). Microscopic examination of thin paraffin sections showed that, compared with fertile anthers, sterile anthers began to show abnormalities at the tetrad stage in which tapetal cells were highly vacuolate (Fig. 1B, F2 and S2). In later stages, tetrads could not develop into mononuclear microspores (Fig. 2H,I), and some became abortive microspores without viability (Fig. 2K). The four anther locules in sterile plants had sequentially shrunk and shriveled with the late pollen developmental stage, which led to pollen sterility (Fig. 2L).

There is an important link between pollen development and the deposition of callose 20. The process of callose deposition is regulated by many factors. Changes in some of these factors lead to abnormal deposition of callose. Low deposition, premature degradation or delayed degradation of callose leads to abnormal pollen development and causes abnormal pollen fertilization and subsequent pollen abortion 47. The blue fluorescence of aniline blue was used to detect the synthesis and degradation during microspore abortion of callose in a male sterility NIL of Chinese cabbage. The misshapen form of many tetrads at the tetrad stage (Fig. 3, S2) and the fact that the tetrads in sterile microspores still exhibited a fluorescence response after tetrad phase (Fig. 3, F3 and S3) suggested that delayed callose degradation could be the important factor that determines pollen sterility in the Chinese cabbage line ‘10L03’.

To determine the main reason for the delayed degradation of callose, we performed a genome‐wide analysis of the GSL and endo‐1,3‐β‐glucosidase enzyme families in Chinese cabbage. Callose biosynthesis is catalyzed by GSL enzymes, which are located on the plasma membrane with the substrate on the cytoplasm side 48. The callose synthesized is deposited between the plasma membrane and the cellulose of the cell wall 20. Previously, 12 GSL genes (*AtGSL1*–*AtGSL12*) were found in *Arabidopsis* 22 and identified to participate in the synthesis of callose 49. Callose is a glucan that contains β‐1,3 bonds 16, and 1,3‐β‐glucosidase enzymes hydrolyze β‐1,3‐glucan (callose), which is widely present in viruses, bacteria, fungi 50, 51 and seed plants 52. *In vitro* experiments confirmed that endo‐1,3‐β‐glucosidase enzymes play a major role in the process of microspore release 45. Stieglitz and Stern (1977) studied the development of microspores in *Lilium* and found that 1,3‐β‐glucosidase enzymes had a peak before the microspores were released from the tetrad, and then the callose walls of the microspores were dissolved to release mature pollen 53. It has been demonstrated that endo‐1,3‐β‐glucosidase enzymes play a key role in this process. However, the mechanism by which this enzyme is accurately induced before microspore maturation is not known 13. The known endo‐1,3‐β‐glucosidase enzymes belong to the seventeenth family of glycosyl hydrolases and their members share a common amino acid sequence structure: (LIVM)‐X‐(LIVM‐FVW)‐(STAG)‐E‐(ST)‐GW‐P‐(ST)‐X‐G 52.

In this study, based on conserved domains and sequence similarities to known *Arabidopsis* callose GSL and endo‐1,3‐β‐glucosidase enzymes, a genome‐wide database were searched, revealing 15 GSL and 77 endo‐1,3‐β‐glucosidase enzymes in the Chinese cabbage genome (Tables 1 and 2). The *B. rapa* has undergone the γ triplication (135 MYA) and the β (90–100 MYA) and α (24–40 MYA) duplications that previous studies revealed 54. In the evolutionary history of Chinese cabbage, three polyploidization events occurred and led to chromosomal reduction, rearrangement, and numerous gene losses. As a result, highly complex gene families have evolved. In this study, the 15 GSL and 77 endo‐1,3‐β‐glucosidase members all clustered closely with their *Arabidopsis* orthologs. Many GSL and endo‐1,3‐β‐glucosidase enzymes in *Arabidopsis* also had two or more counterparts in Chinese cabbage. This result showed that the expansion of the GSL and endo‐1,3‐β‐glucosidase families in Chinese cabbage may have been caused by genome duplication events, including transposition events, tandem duplication, multiple segmental duplication, and entire‐genome duplication 55, 56.

Polyploidization events also cause structural and functional domain diversification. By comparing intron/exon numbers, lengths, distribution and other genetic structures of a gene family, we can realize the diversity of genetic structure 41. In coding region *BraA07g023720* contained the largest number of introns (57). Structural diversification may be caused by a large number of introns (Fig. 4B). Correspondingly, *BraA07g023720* had the largest protein length (Table 1). Variations in intron lengths also occurred in the endo‐1,3‐β‐glucosidase enzymes (Fig. 5). The structural diversity of endo‐1,3‐β‐glucosidase family members in Chinese cabbage provides an evolutionary mechanism for gene duplication, an important step that with exon loss or gain generates structural diversity and complexity 42. The longest exon length was found in *BraA01g003770* relative to other endo‐1,3‐β‐glucosidase enzymes in Chinese cabbage, which indicates that gain of exons might have resulted in functional diversification. As shown in Fig. 5, most endo‐1,3‐β‐glucosidase enzymes had 14 motifs. This difference in motif composition among endo‐1,3‐β‐glucosidase enzymes may potentially have caused functional divergence among different genes. The polyploidization events that occurred in the evolution of the *B*. *rapa* were very important and gave the plant the ability to diversify and respond to changing habitats. New functions have developed for the genes generated from polyploidization events 54.

The large size of these two gene families in Chinese cabbage indicate their importance in the regulation of cabbage‐specific processes. Here, the expression profiles of the 15 GSL and 77 endo‐1,3‐β‐glucosidase genes in different tissues showed that different genes were different expression levels in different organs (Fig. 6A,B). By contrast, *BraA09g010050* and *BraA05g012120* showed peak transcript levels in flowers. Similarly, among the 77 endo‐1,3‐β‐glucosidase genes in Chinese cabbage, *BraA04g003430* and *BraA09g008430* were only expressed and showed peak transcript levels in the flower. Additionally, *BraA03g025370*,* BraA05g031250* and *BraA05g042280* showed high levels of expression in the flower. Therefore, these genes may play a potentially important part during flower development.

The qRT‐PCR results for Chinese cabbage flowers indicated that the synthetic enzyme *BraA01g041620* was highly expressed in the tetrad and meiotic stages in sterile plants (Fig. 7). The phylogenetic results showed that *BraA01g041620* was homologous to *A. thaliana AtGSL9* (Fig. 4). The function of ATGSL9 remains largely unknown, but ZmGSL9 regulated lateral root formation 57. *BraA01g041620* and *AtGSL9* may participate in flower development. *BraA01g041620* may cause excess callose deposition in the meiotic and tetrad stages so that callose degradation is delayed from the tetrad stage. The degrading enzymes *BraA04g008040*,* BraA07g009320*,* BraA01g030220*,* BraA03g040850*,* BraA10g020080* and *BraA05g038340* had low expression in the meiosis and tetrad stages in sterile plants (Fig. 8), among which *BraA04g008040*,* BraA07g009320*,* BraA01g030220* and *BraA03g040850* are homologous genes to *Arabidopsis AT4G14080* (*A6* gene) (Fig. 5). The *A6* gene has been cloned in *A. thaliana* and *B. napus* and is localized in tapetum cells 21. It is expressed at high levels just before the disintegration of the tetrads, and then quickly disappears 14. The callose deposition was abnormal during the release of mutant microspores. The transcription factor *AtMYB103* regulates the *A6* glucanase enzyme, and the loss of *AtMYB103* causes abnormal callose degradation and pollen wall formation 58. In *B. napus*,* BnMs3* influences the expression of *BnA6* to regulate the development of the tapetum, affect the deposition and degradation of callose, and ultimately regulate pollen development 12. We believe that the spatiotemporal specificity of the synthesis and degradation of the callose walls in tetrads are important, as early or delayed degradation causes abnormal pollen development, eventually leading to plant sterility. The low expression of the homologous genes (*BraA04g008040*,* BraA07g009320*,* BraA01g030220* and *BraA03g040850*) of the *Arabidopsis* callose‐degrading enzyme *A6* in sterile plants in the tetrad and meiosis stages would result in delayed degradation of callose. In addition, cluster analysis showed that two other genes (*BraA10g020080* and *BraA05g038340*) also had the same expression trend and may play the same role.

In conclusion, we found that delayed callose degradation from the tetrad stage may be the main cause of abortion in the Chinese cabbage NIL ‘10L03’ with nuclear sterility. Fifteen and 77 members of the callose GSL and endo‐1,3‐β‐glucosidase enzyme families were identified in Chinese cabbage. Relative gene expression analysis of flowers at different developmental stages in Chinese cabbage showed that the high expression of the synthetic enzyme *BraA01g041620* and low expression of the *AtA6*‐homologous genes (*BraA04g008040*,* BraA07g009320*,* BraA01g030220* and *BraA03g040850*) and two other genes (*BraA10g020080* and *BraA05g038340*) for degrading enzymes in the meiosis and tetrad stages may be the cause of the nuclear sterility in NIL ‘10L03’. Overall, our study provided a view of the potential roles of callose GSL and endo‐1,3‐β‐glucosidase enzymes in regulating pollen development in Chinese cabbage. Further research on the synthesis and degradation functions of callose GSL and endo‐1,3‐β‐glucosidase enzymes is needed to determine their roles in the plant and to elucidate the underlying sterility mechanism.

## Conflict of interest

The authors declare no conflict of interest.

## Author contributions

Designed the experiments: YLY and YPY. Performed the experiments: YNP, LYH, YQG and IKR. Analyzed the data: YLY and YNP. Contributed reagents/materials/analysis tools: LYH, YQG and IKR. Wrote the paper: YNP.

## Supporting information


**Fig. S1.** Chromosomal locations of the callose GSL and endo‐1,3‐β‐glucosidase genes in Chinese cabbage.
**Table S1.** Callose GSL enzyme primers used for qPCR analysis.
**Table S2.** Callose endo‐1,3‐β‐glucosidase enzyme primers used for qPCR analysis.
**Table S3.** The meme results of callose GSL enzymes in Chinese cabbage.
**Table S4.** The meme results of callose endo‐1,3‐β‐glucosidase enzymes in Chinese cabbage.
