# Supplementary material for: Genome‐wide analysis of the callose enzyme families of fertile and sterile flower buds of the Chinese cabbage (Brassica rapa L. ssp. pekinensis)
Source: FEBS Open Bio. 2019 Jul 12;9(8):1432–49. doi: 10.1002/2211-5463.12685 (PMC6668379; doi:10.1002/2211-5463.12685)
Supplement: Supplementary file 1 — Fig. S1. Chromosomal locations of the callose GSL and endo‐1,3‐β‐glucosidase genes in Chinese cabbage. Table S1. Callose GSL enzyme primers used for qPCR analysis. Table S2. Callose endo‐1,3‐β‐glucosidase enzyme primers used for qPCR analysis. Table S3. The meme results of callose GSL enzymes in Chinese cabbage. Table S4. The meme results of callose endo‐1,3‐β‐glucosidase enzymes in Chinese cabbage. [file FEB4-9-1432-s001.docx]

**Supplementary Table and Figure**

**Table S1. Callose GSLs enzyme primers used for qPCR analysis.**

| Gene name | Forward primer for qRT-PCR (5'-3') | Reverse primer for qRT-PCR (5'-3') | Tm | amplicon length(bp) | amplicon location(start,stop) |
| --- | --- | --- | --- | --- | --- |
| BraA09g002650 | GTTCCCGTAGCGTTCATGTC | ACCACCTCGACTCTCTCTTCT | 59.02, 59.61 | 165 | 5197, 5361 |
| BraA09g010050 | CAAAGCATCACGCGGCATAA | ACTCAATGTTTGCTCCCCGT | 59.96, 59.91 | 184 | 4218, 4401 |
| BraA10g004390 | CTTACAGGGGCGTTGTGACT | ATCTTCTGCCACTCGAAGCC | 60.03, 60.12 | 115 | 5021, 5135 |
| BraA05g012120 | GGCAATCTCGGAGAGGCTAT | AAGCCTGTGTGCCTTCTCAA | 59.03, 59.82 | 156 | 79, 234 |
| BraA02g004600 | TGGGAAGACCCTCCAGGATT | AAACCGGCGTAACATAGGGA | 59.88, 59.10 | 153 | 1741, 1893 |
| BraA07g023720 | GACGGACAAATGCTACGCTC | CTCGTTTATCCCGCCTGTGA | 59.35, 59.83 | 122 | 33, 154 |
| BraA09g063900 | CCCGTTCGTGTCTGAGTTCC | TTGCCACTCTTGTTCCCACC | 60.67, 60.47 | 171 | 5652, 5822 |
| BraA05g008500 | TTTACCCAGCCCGAATGACC | CCTGTCCCGGTTAATGGCTT | 60.03, 60.03 | 145 | 891, 1035 |
| BraA05g032510 | AGAAGATTCGCCGGAACGAG | GCAAAGGTAAGCGATGCGAG | 60.18, 59.97 | 175 | 75, 249 |
| BraA03g033140 | GGGAGGATGTGGGTGGAAAG | AGGATTCCCAAGAGTGCAGC | 60.03, 60.03 | 131 | 2710, 2840 |
| BraA05g038460 | GACAGACGTTAGCTAGAACAGGA | AATCCGTCGCGTCATTACCA | 59.56, 59.83 | 108 | 3416, 3523 |
| BraA01g041620 | ATGTTGGCCGTGGCATGTAT | TCTTGACACAAAATCATGCCAGTAA | 60.40, 59.47 | 185 | 1922, 2106 |
| BraA09g025290 | AGAGTGGGGAAGACAGGGTT | GTCAACAACGCAACCTCCAC | 60.14, 60.08 | 170 | 856, 1025 |
| BraA10g005220 | ACGTGTTAATGATCCGAAGCG | GATGCTGAGGGAAGGTAGCC | 59.08, 59.89 | 195 | 3360, 3554 |
| BraA10g025720 | TGAAGTCGTTCCCTCATCCC | GAACGCATAGAACCGACAGAG | 59.10, 58.81 | 109 | 108, 216 |
| Tubulin | AGGCGTGTGAGTGAGCAGTT | CATCTCGTCCATTCCTTCACCTGT | 60.22, 59.96 | 146 | 133, 280 |

**Table S2. Callose endo-1, 3-beta-glucosidase enzymes primers used for qPCR analysis.**

| Gene name | Forward primer for qRT-PCR (5'-3') | Reverse primer for qRT-PCR (5'-3') | Tm | amplicon length(bp) | amplicon location(start,stop) |  |
| --- | --- | --- | --- | --- | --- | --- |
| BraA08g009700 | CGTATTGGGCTCAGTTCCGT | TGCCACATGAACCGTGACAA | 60.11, 60.46 | 147 | 1325, 1471 |  |
| BraA05g015330 | GGATCATGTGACAGTGTAAGGG | CAGGCGTTCTTCCTCACAGA | 60.06, 60.18 | 102 | 1462, 1563 |  |
| BraA03g033200 | CGGTGGGACTTGCTCGTTTA | CGGATTGGGAGGATAGAGTGG | 60.32, 59.38 | 118 | 1293, 1410 |  |
| BraA04g008040 | CTCGGTTCCAGCATCAGCAT | CGCGTCGTAGAGCTTGACAT | 60.46, 60.52 | 167 | 65, 231 |  |
| BraA01g030220 | TAAAGCAGGCCACGTTAAGC | GTGAGGAAGGACGTTGGTGT | 58.84, 59.89 | 166 | 183, 348 |  |
| BraA07g009320 | GTTCCTCTTCACTCTCATTGTCC | TTGACGTGGCCTGCTTTTAG | 58.75, 58.77 | 177 | 18, 194 |  |
| BraA03g040850 | ACAAGCAAACCTGGTCGAGT | ACGGTAACATCACCAGAGCG | 59.82, 60.11 | 180 | 428, 607 |  |
| BraA05g038340 | TTACGACGCCAACCCATCAA | AGGAGGTAGCGGATCTTGGT | 59.96, 60.03 | 174 | 165, 338 |  |
| BraA02g013450 | CGGATCAGAGCTACACCGAC | ACGAGATCTTGATCGCTCCG | 59.97, 59.69 | 198 | 269, 466 |  |
| BraA10g014510 | TATCCCGACCAGACGAGTGT | CAGCTCTCACTCCTCCCTCT | 60.03, 60.03 | 104 | 961, 1064 |  |
| BraA07g007140 | GAAGGATCGGCTACAGGGTG | CGCCTCGAGTGTATCTGGAC | 59.89, 59.97 | 109 | 1179, 1287 |  |
| BraA08g018600 | TACCAGCAGAGTGGAGGGAG | ACCAGCCATTGTTCCATCAGT | 60.32, 59.92 | 129 | 1291, 1419 |  |
| BraA03g013540 | CGCTGATTGCCGTTCGATTC | TTCCCGTACTTGGGAGGTTG | 60.32, 59.31 | 171 | 1188, 1358 |  |
| BraA10g013180 | CTCTGTAACGAAGATTGCGCT | GTAGTGCTGCCATAAGGGGT | 58.74, 59.45 | 149 | 1440, 1588 |  |
| BraA01g008280 | ACTGGGGGATGTTCTATGCG | AGGACTTGAGCTTGGACCAG | 59.53, 59.31 | 122 | 989, 1110 |  |
| BraA02g016980 | ACAAATCCGACACATCCGCT | TGAGTTGGACTGACCGATGC | 60.04, 60.04 | 127 | 42, 168 |  |
| BraA02g034940 | CCACACAGGCATCCAACTCA | CTGCGTTGGGTAAGGAGGTT | 60.25, 59.96 | 176 | 198, 373 |  |
| BraA09g010430 | AGCCTAGGAGGAGCGATGAA | GAGGGGCTGAAGACGAGAAC | 60.11, 60.11 | 130 | 1387, 1516 |  |
| BraA08g031380 | CTCCCTTGCTCTTACCAGCC | GGAGATAACCGAGAGCCACG | 60.11, 59.97 | 164 | 449, 612 |  |
| BraA09g006190 | GAAGTCATCTCCACCGCCAA | TGTCGTAGCCTTGTCGGAAC | 60.04, 60.04 | 184 | 394, 577 |  |
| BraA05g006430 | CAGGCCAAGTCCCCATTCAT | GCCATAGCCGAGAGCATTCA | 60.03, 60.25 | 186 | 577, 762 |  |
| BraA07g022070 | CCGCCGTACTTCTCACCTTT | GCGAAGGCACGGAGGATATT | 60.04, 60.25 | 169 | 44, 212 |  |
| BraA04g004630 | GTGCTTTAACCCGAACAGCG | CCTACGACCACACCAGTTCC | 60.11, 60.04 | 117 | 1206, 1322 |  |
| BraA03g058860 | GTGGCGGAAACGGTGCT | CCGCGTCTACCTGTGAAC | 60.67, 57.84 | 100 | 1468, 1567 |  |
| BraA09g046250 | TCGTGCTCTAACCCGAACAG | CCCTACGATGACTCCGCTTC | 59.76, 59.97 | 120 | 1207, 1326 |  |
| BraA01g003770 | GGTTCTAGCGACTCGGGTTC | AGACTCGGTTCAAGCAGACG | 60.18, 60.04 | 145 | 7, 151 |  |
| BraA03g048190 | CAATGTCGACTACCACTGCG | ATCGCCAACATCAAAATTGTCCA | 59.01, 59.74 | 103 | 1061, 1163 |  |
| BraA05g033550 | AAGGGTGTTGGTGGGACTTG | CGTTTGCGTTCCCTCCTACT | 60.11, 60.04 | 166 | 1330, 1495 |  |
| BraA08g012890 | ACCTGGTCCTATTTCGGAGA | AATTGCTTTGTACCTTAGTGGC | 57.46, 57.22 | 155 | 984, 1138 |  |
| BraA01g036400 | TGGTTCGCTCTACTTTCTTTCTCT | CGGCTATGCGACCGTAGTTT | 59.72, 60.53 | 102 | 32, 133 |  |
| BraA09g032320 | CACCTGCATCACCATCGGTA | GATGTCTGGAGGATGGCGAG | 59.82, 59.97 | 165 | 339, 503 |  |
| BraA09g046240 | GGTGTGTGGCTAAGTCGGAA | TAGGGTCAAAGCAAGCACCA | 59.97, 59.52 | 120 | 1106, 1225 |  |
| BraA03g025090 | CTGCTTTGGTCAGTCTCGGT | AATGATCCGGACGAAGGAGG | 59.97, 59.25 | 100 | 425, 524 |  |
| BraA07g020840 | CGAAGTCCTCACCTCCAACC | GATACGTCGAGGATGGCGAG | 60.04, 60.11 | 144 | 354, 497 |  |
| BraA07g018180 | ATCATACATGGCCACACGCA | GGCCGTAGTTGATTCCCACA | 60.11, 60.04 | 116 | 21, 136 |  |
| BraA01g009790 | GGCAATGAATAGGGCCACGA | AGATCGTCCACTCCAATGCC | 60.47, 59.82 | 100 | 1119, 1218 |  |
| BraA06g044140 | GTGGGAAACGAGGCAACAAC | TATTTTGCCGCCGAGAGAGG | 59.97, 60.18 | 111 | 346, 456 |  |
| BraA03g025370 | TGTGGAACCAGATTCTCGCAA | AAGGCTTGGAGGACAGTGTG | 59.93, 59.89 | 169 | 8, 176 |  |
| BraA07g024880 | GGTTGTGTCGGAGATTGGGT | ACCCCCAATGTTTCTCCACG | 59.96, 60.25 | 194 | 852, 1045 |  |
| BraA01g020550 | GTGGATCTCTTCCCGTCGTC | TCTTTGGCGTCCCTCTGTTC | 59.90, 59.97 | 135 | 749, 883 |  |
| BraA02g008840 | CATGACCACGCCGCTTATTG | AGTTCTGTTCAGTCCCTGGC | 59.97, 59.60 | 158 | 825, 982 |  |
| BraA02g008950 | TTTACCGTTGGTGGTGTCCG | TTTGACCTCTTTGGCGTCCC | 60.53, 60.54 | 138 | 765, 902 |  |
| BraA10g020080 | GCTGTTCCTGTTCTTGCTTTCA | GAGGCCGTAGTTTAAGCCGA | 59.64, 59.83 | 106 | 24, 129 |  |
| BraA07g008730 | AGACAAGAACGCTAACGCGA | ATGCCTCTCAAAAGTCCCCG | 60.04, 60.04 | 172 | 855, 1026 |  |
| BraA10g020220 | GTAGTGGCTTCGTAACCGCA | CGCGTAAGGCATCAAGAACC | 60.39, 59.63 | 162 | 71, 232 |  |
| BraA09g047880 | TCTGGGGGATGTTTCTTCCT | TATTGCATCGCTTGCCCTT | 57.94, 57.81 | 126 | 896, 1021 |  |
| BraA10g021890 | TGCAGAATCAGAAGGCGGAG | AATGCCCTGACGTTGGATCA | 60.11, 59.67 | 120 | 1292, 1411 |  |
| BraA01g006680 | CGGGGAACTTTGAGAGGCAT | CCGCGAGAACACACCATTTC | 60.04, 59.83 | 138 | 971, 1108 |  |
| BraA07g022770 | TTCCGTTACATCTCGGTCGG | CGAAGGCGGAAACCCACTAA | 59.55, 60.32 | 159 | 352, 510 |  |
| BraA09g047910 | TATGCTACGGGATGTCAGGC | CATCTGCGTCTGGAGCGTAA | 59.32, 60.18 | 108 | 68, 175 |  |
| BraA04g003380 | TTACGCGGCACTGGAGAAAT | CGCATTCGGCACACTTGTC | 60.04, 60.15 | 103 | 690, 792 |  |
| BraA01g019430 | AGGAGTTTACCAGTATCGCCG | GGCTGCGAGAAGAAAGGAGA | 59.59, 59.75 | 131 | 5, 135 |  |
| BraA02g011800 | CTAGCTGTGGCGCTTGTTTG | CTCTCTCAGCATACGCACCA | 60.11, 59.54 | 108 | 28, 135 |  |
| BraA10g016100 | ACAAGCCAAAGGTGTGAGGT | CCATCCAGATTCCCACACGA | 59.74, 59.46 | 159 | 1056, 1214 |  |
| BraA03g056410 | ACGCACGGCGGTATAATCAA | TCCCTGGCTGAATGCTCTTG | 60.18, 60.03 | 132 | 845, 976 |  |
| BraA09g034950 | AGGACCAACGTCTGAGAGGAA | TCCTTTATAGCCGTGGAGGCA | 60.48, 60.97 | 159 | 966, 1124 |  |
| BraA05g042280 | ATCGTCAACTTCCTCGCACA | TAGCGTCGAAGACGTTCGTG | 59.68, 60.45 | 163 | 589, 751 |  |
| BraA10g016350 | CGATACTTCCTGGTGGGTCG | GCAAAGTTCACGTTCCCACA | 59.90, 59.26 | 181 | 1244, 1424 |  |
| BraA09g011880 | TAAGTGACCCCGTCCCATCT | CCGTCGAAGAAGGCGTAGTT | 59.96, 60.11 | 172 | 503, 674 |  |
| BraA09g008430 | AAGGACGCAAGATCGTACCC | TAAAGCATGGCGGTGCAGT | 59.82, 60.30 | 151 | 1049, 1199 |  |
| BraA04g003370 | CTCAGTTTACGCGGCATTGG | ATAAGTCCCCGCATTCCCCT | 59.90, 60.70 | 118 | 741, 858 |  |
| BraA09g046540 | AAGCAAGGGGTCAGGAACTG | ACCACCTCCACAAAGAACCC | 59.89, 59.81 | 128 | 985, 1112 |  |
| BraA05g031250 | CCTCAACTACGCCCTCTTCG | TGCCAGTGTATCGGCACTTT | 60.18, 59.96 | 147 | 516, 662 |  |
| BraA02g044780 | CGACGGTAAAGGCGAGTCAA | TCCCGGCTTTCTTCAACGAG | 60.39, 60.32 | 101 | 675, 775 |  |
| BraA07g038740 | AAAGACCCCAACAACCAGCA | CTCTCCGACACCACGACTTC | 60.03, 60.11 | 116 | 619, 734 |  |
| BraA04g003430 | AGTAATTCCGAAGCTGTGGCT | CTCCGTTTGGCTATCTGCGA | 59.72, 60.18 | 165 | 133, 297 |  |
| BraA03g009860 | CACAAGCGAGCCATCCAATG | GGACGCCATAGATGCCAAGA | 59.90, 59.89 | 173 | 107, 279 |  |
| BraA04g003410 | CAAGGACGACAACAACCAGC | AGATCCATTCGCTCTCCGA | 59.69, 57.88 | 128 | 672, 799 |  |
| BraA02g009170 | AAGGGCGTTCGTTACCAGG | GGTCGACGCTCGGCAAG | 60.00, 60.89 | 105 | 1087, 1191 |  |
| BraA07g022860 | TCTGCGACAAGCAACAAAGC | ACTGCAACAGGTCAGAGTGG | 59.97, 59.89 | 194 | 968, 1161 |  |
| BraA02g024430 | GTAATGAGCGTTGCAGGGGA | TCTCTTCGTTTCGTGGACCG | 60.39, 60.04 | 199 | 77, 275 |  |
| BraA04g003450 | GTCCGCAACTACACAGAGGG | ACCGCGGGTGTCTATAGTTG | 60.39, 59.54 | 165 | 556, 720 |  |
| BraA09g047900 | ACGACGGTGTCAGATTCAGG | CGACACCTTGATCCCCATCC | 59.76, 60.18 | 137 | 152, 288 |  |
| BraA04g003400 | TTGGAAGTCGTGGTGTCGG | TCGAACATGGCGAATATGTTGTATC | 59.93, 59.82 | 114 | 403, 516 |  |
| BraA04g003390 | CCTCGGGGTCAAGTTTGGAG | CTCCAGTGCCGCGTAGATAG | 60.32, 60.04 | 122 | 471, 592 |  |
| BraA04g003420 | ATCCGTAACAACGTCCGCAA | GGAGAAACATCGCGTTTGGG | 60.32, 59.83 | 100 | 259, 358 |  |
| BraA02g045500 | TTTGCAGGAGCTGGTCAAGAA | GGTAACGAGAGAGGACATGGG | 60.13, 59.59 | 181 | 46, 226 |  |
| Tubulin | AGGCGTGTGAGTGAGCAGTT | CATCTCGTCCATTCCTTCACCTGT | 60.22, 59.96 | 146 | 133, 280 |  |

**Table S3.The MEME results of Callose GSLs enzyme in Chinese cabbage.**

| **MEME number** | **gene** |
| --- | --- |
| 20 | ALL Callose GSLs enzyme in Chinese cabbage |

**Table S4. The MEME results of Callose endo-1, 3-beta-glucosidase enzymes in Chinese cabbage.**

| **MEME number** | **gene** |
| --- | --- |
| 14 | BraA04g003430; BraA07g038740; BraA04g003410; AT3G57260; BraA09g047880; BraA09g047910; BraA07g022770; AT3G57240; BraA01g019430;BraA01g020550; AT4G16260; BraA10g020220; BraA10g020080; AT5G20390; BraA02g008840; AT5G20330; AT5G20340; BraA06g044140; BraA06g044140; BraA03g048190; BraA08g012890; BraA01g009790; BraA09g034950; BraA09g032320; AT1G32860; BraA07g020840; AT5G42100; BraA07g024880 |
| 15 | BraA04g003380; BraA04g003370; AT3G57270; BraA10g021890; BraA09g046250; BraA01g036400; BraA03g025370; AT2G27500; BraA07g018180; BraA03g025090; |
| 16 | BraA09g011880; AT1G64760; BraA05g006430; BraA07g022070; BraA04g004630; BraA09g046240; BraA09g006190; |
| 17 | BraA05g042280; BraA09g008430; BraA02g044780; BraA03g058860 |
| 18 | BraA10g016350; AT5G58480; BraA07g008730; BraA03g009860; BraA02g009170; BraA01g006680; BraA03g056410; AT4G31140; AT5G58090; BraA02g011800; BraA10g016100; BraA03g033200; BraA08g009700; BraA05g015330; BraA02g016980; BraA02g013450; BraA10g013180; BraA03g013540; BraA07g007140; BraA09g010430; BraA01g003770; AT4G34480 |
| 7 | BraA04g003400; BraA02g045500 |
| 8 | BraA09g047900; |
| 9 | BraA04g003390; BraA02g013450 |
| 5 | BraA04g003420; |
| 12 | BraA02g024430; AT5G20560; AT1G33220; BraA05g031250; BraA09g046540; |
| 19 | AT3G07320; BraA05g038340; BraA10g020220; BraA07g009320; BraA03g040850; AT4G14080; BraA04g008040; BraA01g008280; AT4G29360; BraA08g018600; BraA10g014510; AT5G56590; BraA08g031380; AT1G11820; BraA05g033550; AT3G13560; AT1G66250; BraA02g034940; AT2G01630 |
| 10 | BraA07g022860; |

**
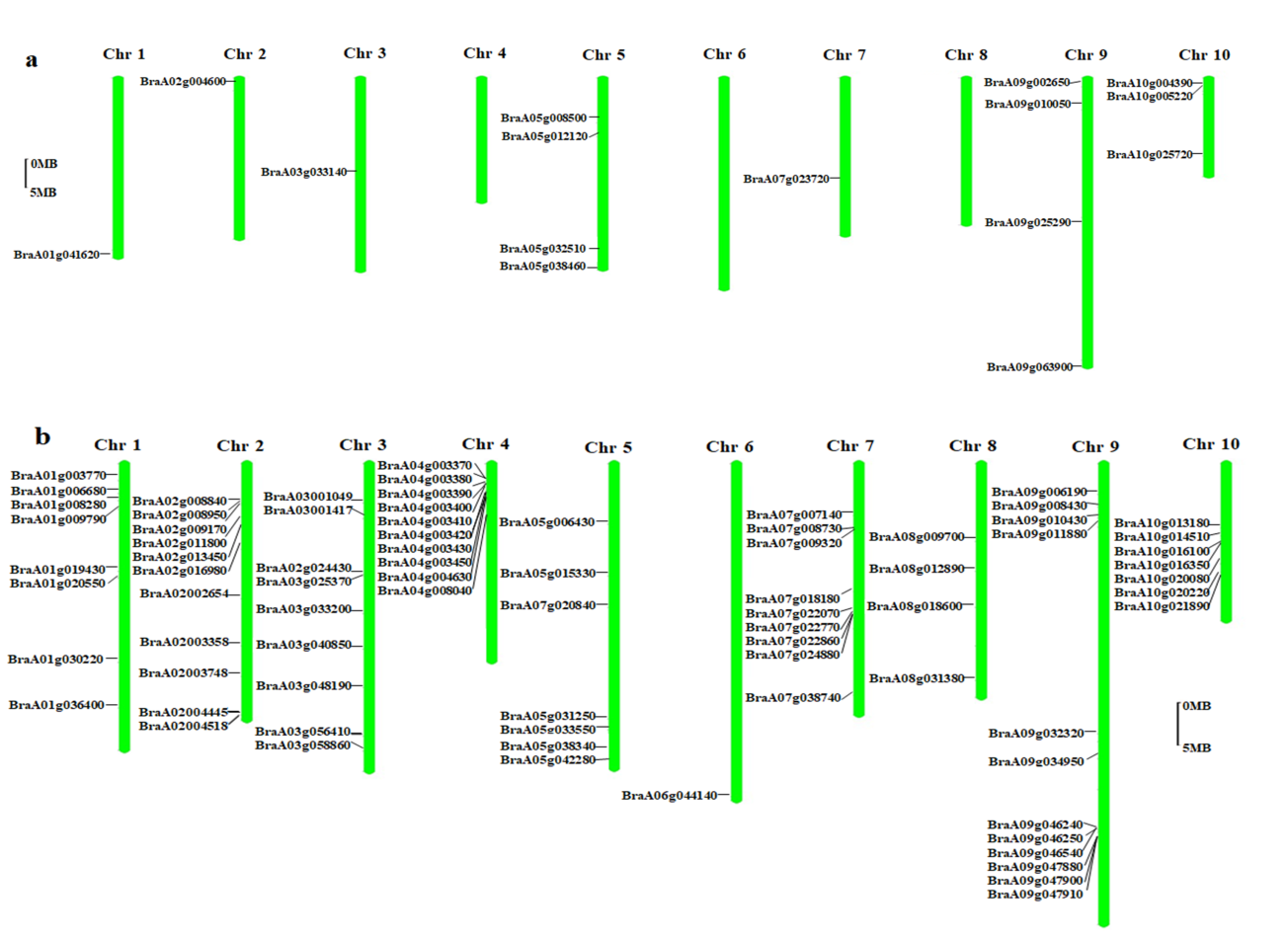
**

**Supplementary Figure 1. Chromosomal locations of the callose GSL and endo-1,3-beta-glucosidase genes in Chinese cabbage.** **(a)** The distribution of callose GSL genes on the 10 chromosomes. **(b)** The callose endo-1,3-beta-glucosidase genes were unevenly distributed among the Chinese cabbage chromosomes. The chromosomal distributions of Chinese cabbage genomic sequences were drawn with the MapInspect tool (http://www.plantbreeding.wur.nl/UK/software_mapinspect.html)
